# In vivo chemical reprogramming of astrocytes into neurons

**DOI:** 10.1038/s41421-021-00243-8

**Published:** 2021-03-02

**Authors:** Yantao Ma, Handan Xie, Xiaomin Du, Lipeng Wang, Xueqin Jin, Qianqian Zhang, Yawen Han, Shicheng Sun, Longteng Wang, Xiang Li, Changjiang Zhang, Mengdi Wang, Cheng Li, Jun Xu, Zhuo Huang, Xiaoqun Wang, Zhen Chai, Hongkui Deng

**Affiliations:** 1 Department of Cell Biology, School of Basic Medical Sciences, Peking University Stem Cell Research Center, State Key Laboratory of Natural and Biomimetic Drugs, Peking University Health Science Center, Beijing, 100191 China; 2 Academy for Advanced Interdisciplinary Studies, Peking University, Beijing, 100871 China; 3 MOE Key Laboratory of Cell Proliferation and Differentiation, College of Life Sciences, Peking-Tsinghua Center for Life Sciences, Peking University, Beijing, 100871 China; 4 Peking University-Tsinghua University-National Institute of Biological Science Joint Graduate Program, College of Life Science, Peking University, Beijing, 100871 China; 5 State Key Laboratory of Membrane Biology, College of Life Sciences, Peking University, Beijing, 100871 China; 6 Department of Molecular and Cellular Pharmacology, School of Pharmaceutical Sciences, Health Science Center of Peking University, Beijing, 100871 China; 7 State Key Laboratory of Brain and Cognitive Science, CAS Center for Excellence in Brain Science and Intelligence Technology (Shanghai), Institute of Biophysics, Chinese Academy of Sciences, Beijing, 100101 China; 8 University of Chinese Academy of Sciences, Beijing, 100049 China; 9 School of Life Sciences, Joint Graduate Program of Peking-Tsinghua-NIBS, Peking University, Beijing, 100871 China; 10 School of Life Sciences, Center for Bioinformatics, Center for Statistical Science, Peking University, Beijing, 100871 China; 11 Beijing Institute for Brain Disorders, Beijing, 100069 China; 12 Beijing Advanced Innovation Center for Big Data-Based Precision Medicine, Beihang University & CapitalMedical University, Beijing, 100191 China; 13 Shenzhen Stem Cell Engineering Laboratory, Key Laboratory of Chemical Genomics, Peking University Shenzhen Graduate School, Shenzhen, Guangdong, 518055 China; 14 Present Address: State Key Laboratory of Membrane Biology, Beijing Advanced Innovation Center for Structural Biology, Tsinghua-Peking Joint Center for Life Sciences, School of Life Sciences, Tsinghua University, Beijing, 100084 China; 15 Present Address: The Murdoch Children’s Research Institute, University of Melbourne Department of Paediatrics, Flemington Road, Parkville, VIC 3052 Australia

**Keywords:** Reprogramming, Transdifferentiation

## Abstract

In mammals, many organs lack robust regenerative abilities. Lost cells in impaired tissue could potentially be compensated by converting nearby cells in situ through in vivo reprogramming. Small molecule-induced cell reprogramming offers a temporally flexible and non-integrative strategy for altering cell fate, which is, in principle, favorable for in vivo reprogramming in organs with notoriously poor regenerative abilities, such as the brain. Here, we demonstrate that in the adult mouse brain, small molecules can reprogram astrocytes into neurons. The in situ chemically induced neurons resemble endogenous neurons in terms of neuron-specific marker expression, electrophysiological properties, and synaptic connectivity. Our study demonstrates the feasibility of in vivo chemical reprogramming in the adult mouse brain and provides a potential approach for developing neuronal replacement therapies.

## Introduction

Cell fate control and manipulation is a fundamental process in biology and enables the generation of desired cell types for regenerative medicine. Using chemical approach for reprogramming has become an advance in cell fate manipulation^1,2^. Somatic cells can be reprogrammed into pluripotent stem cells by using small molecules^3–7^. Furthermore, different functional lineages can be generated by chemically induced lineage reprogramming in vitro^8–13^. The small-molecule approach offers unique advantages in cell fate modulation, including high cell permeability, reversibility, and easy manipulation^14,15^, thus making this approach a promising strategy of cell fate manipulation.

In principle, the chemical approach is favorable for in vivo applications. In vivo cell fate reprogramming has emerged as a promising strategy that could compensate for cell loss by converting resident cells in situ^16–21^. It could circumvent difficulties faced by cell transplantation, including cell purification, long-term survival, and functional integration^22,23^. Importantly, the favorable environmental niche in the native tissue potentially facilitates the functional maturation and timely integration of in vivo reprogrammed cells^14,15,22–24^. However, further application of in vivo reprogramming may be impeded by delivery and safety concerns. Small molecules are non-immunogenic, non-integrative to the genome and their manipulation of intracellular targets are reversible. These characteristics favor the potential of small molecules for cell-fate manipulation in vivo to regenerate cells for endogenous tissue repair.

Because of its limited regenerative ability, the mammalian central nervous system is a desirable target for assessing in vivo chemical reprogramming, and neuroglial cells (such as astrocytes) may potentially be used to generate neurons using in vivo reprogramming in situ. Currently, the loss of neurons due to brain trauma or neurodegeneration is irreversible; however, astrocytes are one of the major cell types that respond, proliferate, and assemble to enclose necrotic lesions, making them ideal targets for in vivo reprogramming^25,26^. Therefore, it is important to develop a chemical approach to convert resident astrocytes into functional neurons in situ, which could favor the timely integration of newly generated cells into native neuronal tissue.

In our previous studies, we demonstrated that fibroblasts can be efficiently converted into functional neurons by chemical reprogramming in vitro^11,12^. Here, we developed an in vivo chemical approach that could reprogram resident astrocytes into neurons with synaptic connectivity in the adult mouse brain.

## Results

### A chemical cocktail efficiently converts astrocytes into neurons in vitro

We reported earlier that a chemically defined cocktail, FICB (Forskolin, ISX9, CHIR99021, and I-BET151), could reprogram fibroblasts into functional neurons in vitro^12^. Here we tested the effects of this cocktail on the conversion of astrocytes carrying the neuron-specific TauEGFP reporter into neurons (Supplementary Fig. S1a). Upon exposure to FICB, astrocytes isolated from postnatal day-0–3 mice underwent neuron-like morphological changes, and TauEGFP^+^ cells were observed at 16 days post-induction (dpi), suggesting that neuronal cell fate had been acquired (Supplementary Fig. S1b). An optimized cocktail, DFICBY (DBcAMP, Forskolin, ISX9, CHIR99021, I-BET151, and Y-27632) (Supplementary Table S1), enhanced neuronal conversion efficiency, yielding 91.1% (SEM, 2.6%) of TauEGFP^+^ cells with neurite outgrowth (Supplementary Fig. S1c–e). We further found that culture medium supplemented with bFGF was important for cell survival and neuronal conversion (Supplementary Fig. S1d). These chemical-induced cells also expressed neuron-specific markers TUJ1 and MAP2 at 16 dpi (Supplementary Fig. S1e). To continue maturation, we co-cultured reprogrammed neurons with primary astrocytes in a defined maturation medium for an additional 2 weeks. Subsequently, the reprogrammed TauEGFP^+^ cells expressed synaptic protein SYN1, mature neuronal marker NEUN (94.1% [SEM, 4.1%]), as well as glutamatergic neuronal marker vGLUT2 and GABAergic neuronal marker GAD67 (Supplementary Fig. S1h, i). Whole-cell patch-clamp recording showed that the chemically induced neurons (CiNs) were generating action potentials (APs) and inward currents, suggesting they had acquired electrophysiological functions (Supplementary Fig. S1j and Table S1).

To confirm the astroglial origin of the CiNs induced in vitro, we traced the original GFAP-expressing astrocytes that were isolated from the *Gfap-cre/Rosa26-tdTomato* mice (Supplementary Fig. S2a). Nearly all isolated tdTomato^+^ cells were immunopositive for astroglial-specific markers and negative for markers of other neural lineages, suggesting that tdTomato highly specifically labeled astrocytes (Supplementary Fig. S2b, c). At 16 dpi after DFICBY treatment, we observed that 97.4% (SEM, 1.2%) of TUJ1^+^ cells co-expressed tdTomato, suggesting the TUJ1^+^ CiNs were converted from astrocytes (Supplementary Fig. S2d, e). Among tdTomato^+^ cells, 88.2% (SEM, 2.7%) expressed TUJ1(Supplementary Fig. S2f). Meanwhile, since astrocytes isolated during postnatal days 5–7 were more restricted to glial fate, we also treated those cells with our chemical cocktail and observed no significant difference in conversion efficiency compared with that of P0–P3 cultures (Supplementary Fig. S1f, g). Collectively, these results indicated that the DFICBY cocktail robustly reprogrammed astrocytes into CiNs with functional neuronal properties in vitro.

To understand the transcriptomic dynamics of astrocyte-to-neuron conversion driven by small molecules in vitro, we performed RNA-sequencing at different time-points during the first stage of conversion (D0–D16). When comparing the global gene expression patterns among different samples, we observed that chemically induced cells grouped closely to the primary neurons but were distinct from astrocytes (D0 samples) in the dendrogram plot (Supplementary Fig. S3a). Similarly, principal component analysis showed that chemical treatment-induced astrocytes went through a conversion trajectory towards primary neurons (Supplementary Fig. S3b). We also produced heatmaps and conducted Gene Ontology (GO) analysis of the differentially expressed genes involved in cell fate conversion in vitro (Supplementary Fig. S3c–e). During the first 4 days, we observed a rapid down-regulation of genes involved in glial cell fate commitment and cell proliferation, demonstrating a rapid cell cycle arrest and erasure of astrocyte identity. Meanwhile, we identified a gradual upregulation of genes involved in neuron fate specification, axonogenesis, synapse organization, and electrophysiological functions along with the reprogramming process. Additionally, transcriptional factors and hallmark genes of neuronal subtypes, including GABAergic and glutamatergic neurons, were also upregulated. Some of the mature neuronal functional genes in 16-dpi samples did not reach the same expression levels as those of primary neuron samples, thus indicating the necessity of co-culturing them with primary astrocytes for maturation (Supplementary Fig. S3b, c). Wnt, Notch, BMP, and cAMP-mediated signaling pathways were also identified during the reprogramming process (Supplementary Fig. S3d). Furthermore, we observed elevated levels of proneural genes, including *Scrt1*, *Neurod1*, *Pou4f1*, and *Neurog2*, in this process (Supplementary Fig. S3e, f).

### A further optimized chemical cocktail converts astrocytes into neurons in vivo

Next, we tested whether the chemical cocktail could induce resident astrocytes into CiNs in adult mouse brains. We initially used *Gfap-cre* transgenic mice carrying a *Rosa26-tdTomato* reporter to genetically fate-map astrocytes in the striatum of adult mice^27^. We administered the small-molecule cocktail DFICBY into the striatum of 8-week-old mice for 2 weeks via an osmotic mini-pumping system. After DFICBY induction, we observed NEUN^+^/tdTomato^+^ cells in the striatum at 8 weeks post-injection (wpi), suggesting that this cocktail had the potential to successfully reprogram astrocytes into neuron-like cells in vivo (Supplementary Fig. S4). We further optimized the cocktail for in vivo reprogramming by increasing the dosage of the small molecules to threefold the initial dose that was used during the in vitro conversion (Supplementary Fig. S4a). We also compared the two small molecules with the same target, Forskolin and DBcAMP, and found that a high dosage of Forskolin alone tended to generate more mature CiNs that could fire action potentials (data not shown). The optimized formula, FICBY, was better at generating NEUN^+^/tdTomato^+^ cells in vivo than DFICBY.

To investigate whether each small molecule in the FICBY combination is needed, we serially omitted components from the cocktail and observed the results. The cocktail without either Forskolin, I-BET151, ISX9, or CHIR99021 failed to generate CiNs in vivo, while the absence of Y-27632 significantly reduced reprogramming efficiency (Supplementary Fig. S4b), thus suggesting that the FICBY small molecules synergistically reprogrammed astrocytes into CiNs. In the in vitro reprogramming, we found bFGF was important for cell survival and neuronal conversion; however, the molecule was not indispensable for in vivo reprogramming (Supplementary Fig. S4b), as the in vivo microenvironment might compensate the function of the component, bFGF.

Additionally, no apparent cell apoptosis was found at 1, 2, and 8 wpi (Supplementary Fig. S5a). We rarely found signals of glial scar markers, CSPG, and COL2A1 at the infusion site in both the vehicle and chemically treated groups (Supplementary Fig. S5b). IBA1 staining showed an increase of signal within the first 2 wpi, then decayed to a normal level at 8 wpi, which was similar to that of the intact group. However, we did not observe significant difference in the IBA1 level between the vehicle and chemically treated groups, indicating that a transient microglial response was potentially triggered by the infusion procedure, but not by the small molecules (Supplementary Fig. S5c).

### Chemically induced astrocyte-to-neuron conversion confirmed by conditional lineage tracing in both striatum and cortex

**Fig. 1:**
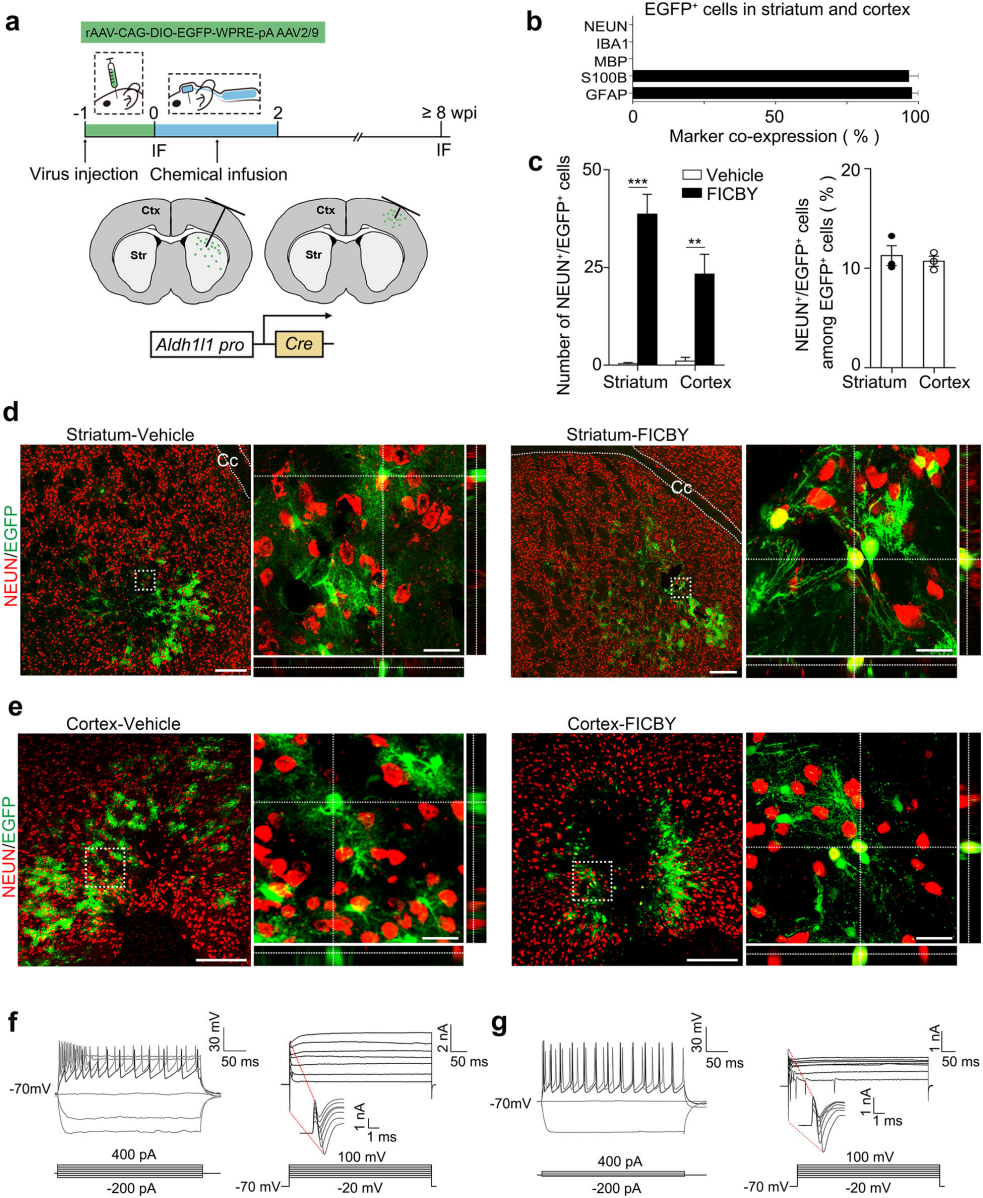
In vivo chemical induction of resident astrocytes into CiNs in conditional lineage-tracing system. **a** Schematic diagram of chemical reprogramming in the striatum or cortex of the *Aldh1l1-cre* mice injected with AAV-FLEX-EGFP. IF, immunofluorescence. **b** Characterization of EGFP^+^ cells in the lineage-tracing mice (*n* = 3 mice). **c** Quantification of NEUN^+^/EGFP^+^ cells from 5 brain sections at 45-μm intervals per mouse in the striatum and cortex, respectively (*n* = 3 mice). Quantification of conversion efficiency of CiNs among EGFP-labeled cells in the striatum and the cortex, respectively (*n* = 3 mice). **d** Immunofluorescence analyses showing NEUN^+^/EGFP^+^ cells in the striatum after chemical treatment was conducted. Cc, corpus callosum. **e** Immunofluorescence analyses showing NEUN^+^/EGFP^+^ cells in the cortex following chemical treatment. **f** Schematic representation of the action potential traces and inward currents of CiNs induced in the striatum (*n* = 9). **g** Schematic representation of the action potential traces and inward currents of CiNs induced in the cortex (*n* = 3). Scale bars, 200 μm; 25 μm in high-magnification panels (**d**, **e**). Error bars represent SEM. **P < 0.01, ***P < 0.001 by two-way ANOVA with Sidak’s multiple comparisons test.

To ensure the astrocyte origin of CiNs, we examined in vivo astrocyte-to-neuron conversion using conditional lineage tracing with *Aldh1l1-cre* mice^28,29^ injected with flexed EGFP-adeno-associated virus (AAV-FLEX-EGFP) (Fig. 1a). This system enabled specific labeling of Aldehyde dehydrogenase 1 family member L1 (ALDH1L1)-expressing astrocytes that were restricted to the striatum and cortex at adulthood, thus excluding the possibility of mislabeling neurons that transiently expressed astroglial genes during development. Additionally, by lowering the volume and titer of injected AAV, we avoided labeling astrocytes in the adult neurogenesis zone, including the subventricular and subgranular zones. EGFP^+^ cells that we observed in both the striatum and the cortex expressed astrocyte markers GFAP and S100B, but not markers of other neural cells, including microglial marker IBA1, oligodendrocyte marker MBP, mature neuron marker NEUN, and neural progenitor markers, including NESTIN and VIMENTIN (pS55) (Fig. 1b; Supplementary Fig. S6), thus suggesting highly specific targeting in astrocytes.

To systemically quantify CiN conversion efficiency, we analyzed 5 brain sections at 45-μm intervals per mouse throughout the rostral-caudal extent on both sides of the cannula. We analyzed the distribution of CiNs in brain sections and found that the majority segregated in a circular area, with the bottom of the infusion cannula as the center point and a 300-μm radius being the effective area of the small molecule infusion site (Fig. 1d, e). After small-molecule treatment, we detected 39 (SEM, 5) and 23 (SEM, 5) NEUN^+^/EGFP^+^ CiNs within the effective area of striatum and cortex, respectively (Fig. 1c–e). However, almost no NEUN^+^/EGFP^+^ cells were detected in the vehicle groups. The conversion efficiency on single section reached 11.3% (SEM, 1.0%) of all the EGFP-labeled cells in the striatum and 10.7% (SEM, 0.5%) in the cortex (radius = 300 μm) (Fig. 1c). Since we could not completely ensure that all labeled astrocytes were effectively treated by small molecules, we may have underestimated the reprogramming efficiency. In addition, using whole-cell patch-clamp recordings, we showed that the CiNs generated in both the striatum and the cortex had the potential to fire continuous APs. Fast, inactivating inward and outward currents were also recorded on the CiNs, indicating mature membrane properties that resembled endogenous neurons (Fig. 1f, g; Supplementary Fig. S7 and Table S2).

**Fig. 2:**
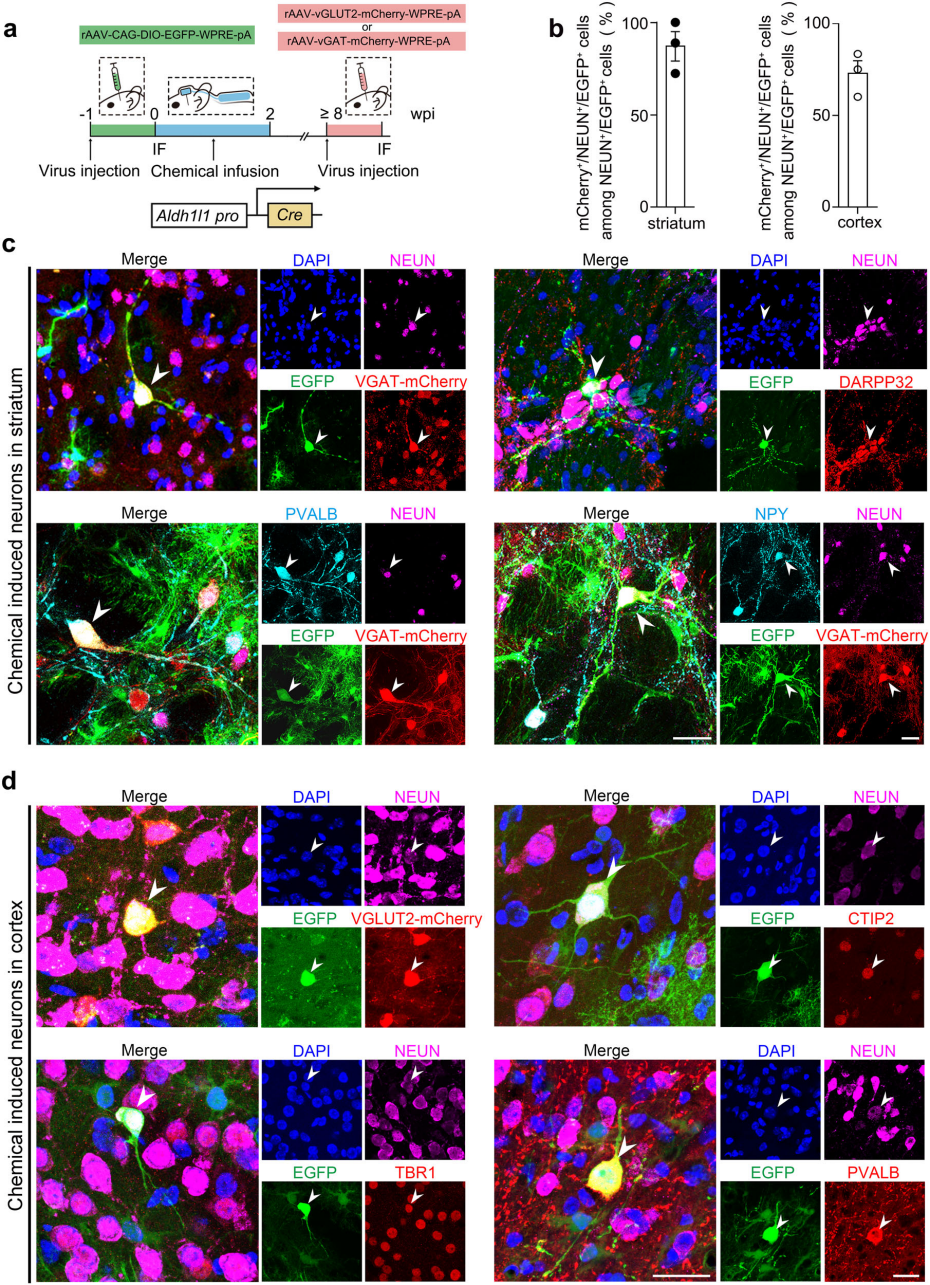
Neuronal subtype characterizations of CiNs in the striatum and the cortex. **a** Schematic diagram of chemical reprogramming in the striatum or the cortex of the *Aldh1l1-cre* mice injected with AAV-FLEX-EGFP and AAV-vGAT-mCherry or AAV-vGLUT2-mCherry sequentially. IF, immunofluorescence. **b** Conversion efficiency showing the generation of the VGAT-mCherry^+^/NEUN^+^/EGFP^+^ cells induced in the striatum and the VGLUT2-mCherry^+^/NEUN^+^/EGFP^+^ cells induced in the cortex (*n* = 3 mice). **c** Striatal neuronal subtype markers, including VGAT-mCherry, DARPP32, PVALB, and NPY were detected in CiNs generated in the striatum of adult mice. Arrowheads show the co-localization of EGFP and the corresponding markers. **d** Cortical neuronal subtype markers, including VGLUT2-mcherry, CTIP2, TBR1, and PVALB were detected in CiNs generated in the cortex of adult mice. Arrowheads indicate the co-localization of EGFP and the corresponding markers. Scale bars, 25 μm. Error bars represent SEM.

Since the striatum is mainly comprised of GABAergic neurons, we used AAV-VGAT-mCherry^30^ to mark the GABAergic neurons following chemical induction and subsequently identified mCherry^+^/NEUN^+^/EGFP^+^ cells as CiNs that resembled GABAergic neurons (Fig. 2a). Among the NEUN^+^/EGFP^+^ cells, we identified 87.1% (SEM, 8.0%) as GABAergic-like neurons, a proportion that resembled the endogenous composition in adult striatum (Fig. 2b). Other striatum-specific neuronal subtype markers, including DARPP32, NPY, and PVALB were also detectable in CiNs (Fig. 2c). In the cortical region, we used AAV-VGLUT2-mCherry^31^ to mark glutamatergic neurons and found that 72.8% (SEM, 6.8%) of the NEUN^+^/EGFP^+^ cells that we analyzed resembled glutamatergic neurons (Fig. 2a, b). We also detected other cortex-specific neuronal markers, including CTIP2, TBR1, and PVALB, in CiNs (Fig. 2d). Collectively, these results suggest that the newly generated in situ CiNs resembled the characteristics of region-specific subtypes.

### Exclusion of aberrant Cre recombination during in vivo chemical reprogramming

To exclude the possibility of mistaken Cre recombination in endogenous neurons during chemical induction, we analyzed whether astrocyte genes would be activated in endogenous neurons during reprogramming process. First, we analyzed over 500 AAV-hSyn-EGFP-labeled endogenous neurons at the core of the reprogramming area in the striatum or cortex (Supplementary Fig. S8a, b). At 1, 2, 4, and 8 wpi, GFAP or ALDH1L1 were not detected in the EGFP^+^ endogenous neurons, indicating endogenous neurons unlikely expressed astrocyte genes during the whole reprogramming process (Supplementary Fig. S8c, d).

Next, to genetically trace astrocyte gene expression in endogenous neurons, we injected AAV-FLEX-EGFP into the striatum of *Aldh1l1-cre* mice. This approach enabled us to label the cells that once or transiently expressed *Aldh1l1*. Meanwhile, we used a specific retrograde neuronal tracer, Alexa Fluor 555-conjugated cholera toxin subunit B (CTB), to label endogenous neurons in the striatum (Supplementary Fig. S9a). Before chemical induction, we confirmed that the areas of CTB labeling and AAV labeling merged well. This ensured that CTB-labeled endogenous neurons were mostly infected with the AAV virus. At 2 wpi, we analyzed over 500 CTB^+^ endogenous neurons at the core of the reprogramming area and detected no EGFP^+^/CTB^+^ neurons, indicating that astrocyte gene would not be activated in striatal neurons during the small molecule infusion period (Supplementary Fig. S9b). At 8 wpi, the density of CTB labeling decreased, which might be caused by cell excretion of CTB. Thus, we analyzed the remaining CTB^+^ neurons and observed no EGFP^+^/CTB^+^ neurons either (Supplementary Fig. S9b). Our observation at 8-wpi suggested that astrocyte gene was unlikely activated in striatal endogenous neurons during the whole reprogramming process.

Finally, we co-cultured TauEGFP^+^ neurons isolated from *Gfap-cre/Rosa26-tdTomato/TauEGFP* mice, with tdTomato^+^ astrocytes isolated from *Gfap-cre/Rosa26-tdTomato* mice. After 16 days of small molecule cocktail treatment, we checked over 500 cells and found no TauEGFP^+^/tdTomato^+^ neurons (Supplementary Fig. S9c). Collectively, these results suggested that an inaccurate Cre recombination were unlikely activated in pre-existing neurons.

### Single-cell RNA sequencing analysis of CiNs in the striatum

**Fig. 3:**
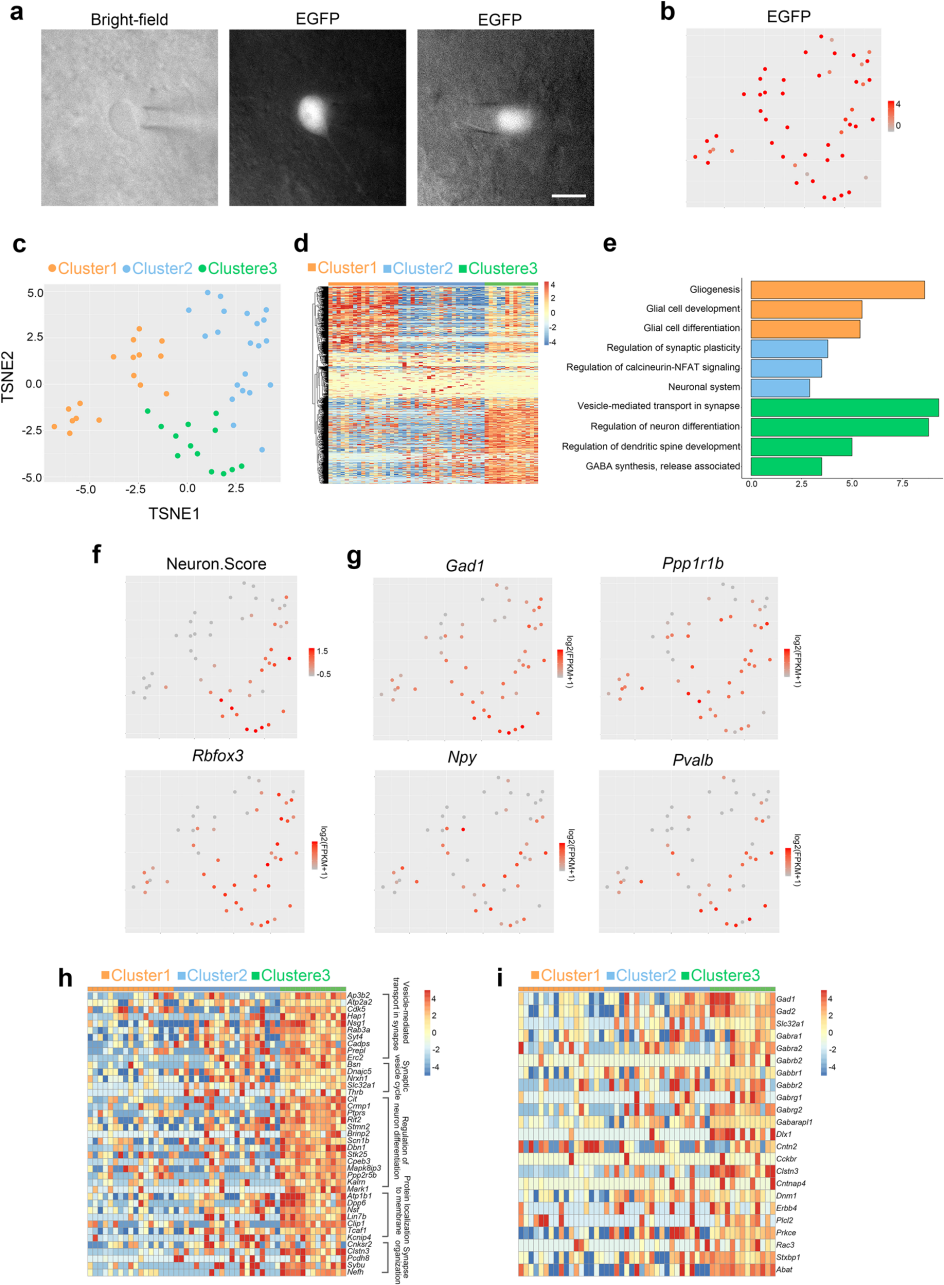
Single-cell RNA sequencing analysis on CiNs in the striatum. **a** Representative images of neuron-like EGFP^+^ cells in brain slices collected by patch pipette. Scale bar, 10 μm. **b** EGFP expression was confirmed in the 51 cells analyzed. **c** t-SNE analysis of highly variable genes that were detected in 51 EGFP^+^ cells. **d** Heatmap representation of 1256 differentially expressed genes cross the three clusters. **e** Enriched GO terms of 1256 differentially expressed genes cross the three clusters. **f** Gene expression profiles of the EGFP^+^ cells using the neuron score analysis. **g** Expression patterns of striatal neuronal subtype genes in the three clusters. **h** A heatmap showing expression levels of genes involved in mature neuronal functions in the three clusters. **i** A heatmap showing expression levels of GABAergic neuronal genes in the three clusters.

Next, we performed single-cell RNA sequencing analysis to further characterize the CiN transcriptome in the striatum of the *Aldh1l1-cre* mice injected with AAV-FLEX-EGFP. Using the patch pipetting technique, we collected EGFP-labeled cells at 12–16 wpi from brain slices (Fig. 3a). We analyzed 14,643 genes across 53 cells, after filtering out low-quality cells and low-abundance genes, and confirmed EGFP expression in 51 cells, of which we analyzed as follows (Fig. 3b). We identified 3 clusters of cells with distinct expression profiles using t-stochastic neighbor embedding (t-SNE) analyses, and characterized the molecular properties of each cluster using heatmap and GO analyses of 1256 differentially expressed genes across the 3 clusters (Fig. 3c–e). Remarkably, cluster 3 showed many GO terms involved in mature neuronal functions, especially GABAergic neuron functions, suggesting that cells in that cluster had transcriptional features of mature neurons. Cells in cluster 2 also showed enrichment of GO terms involved in neuronal functions, including regulation of synaptic plasticity, neuronal system, and calcineurin-NFAT signaling, but they were less than those of cluster 3, suggesting that cluster 2 cells’ neuronal properties were still in the reprogramming process and not yet mature. Compared to clusters 2 and 3, cluster 1 cells enriched some GO terms involved in glial cell development, thus indicating that these cells retained some astrocyte identities.

Notably, the neuronal identities of cells in cluster 3 were confirmed by neuron score analysis (Fig. 3f). Pan-neuronal hallmark genes, including *Rbfox3*, *Nfeh*, and *Sybu*, were highly expressed in cluster 3 cells (Fig. 3f, h). We also found a high-level of expression in genes involved in synaptic activities and GABAergic neuron-specific functions in cluster 3, indicating that CiNs in the striatum developed mature transcriptomic characteristics of GABAergic neuron (Fig. 3h, i). In addition, the expression of striatum-specific subtypes of genes *Gad1*, *Ppp1r1b*, *Npy*, and *Pvalb*, were detected in cluster 3 cells, showing that region-specific CiN characteristics were likely established by in vivo chemical reprogramming (Fig. 3g).

### CiNs received connections from the host neurons

**Fig. 4:**
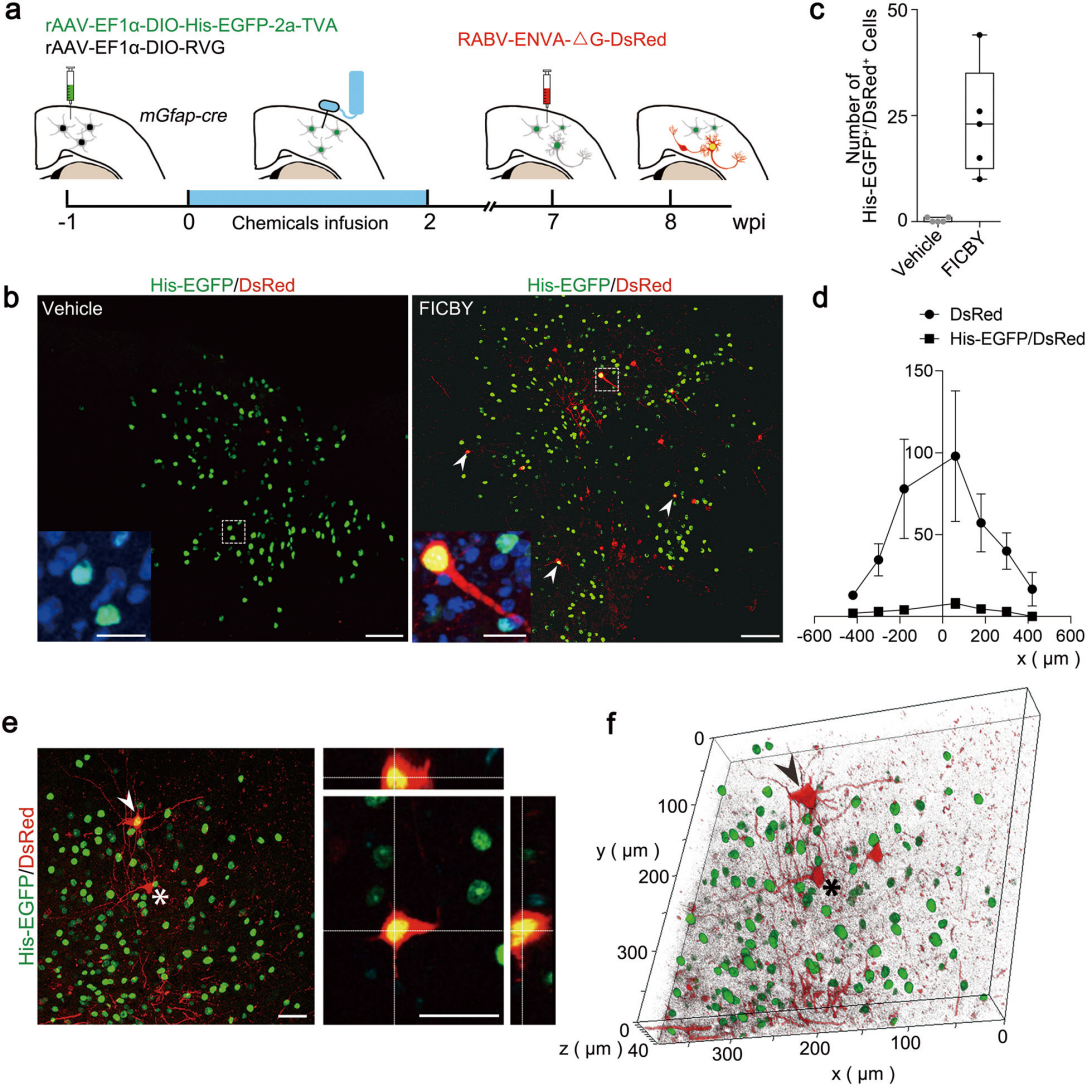
Integration of in vivo reprogrammed CiNs into the mouse brain. **a** Schematic representation illustrating the RABV-based retrograde transsynaptic tracing process. **b** Illustrative images of the initiating astrocytes (green), CiNs (yellow nucleus and red cytoplasm) and traced host neurons (red) in the chemically induced and vehicle groups at 8 wpi. Arrowheads point to the examples of the CiNs. High-magnification panels represent the co-localization of His-EGFP and DAPI. **c** Histogram representing the total number of His-EGFP^+^/DsRed^+^ cells (CiNs) in the chemically induced and vehicle groups (*n* = 5 mice). **d** Quantifications of His-EGFP^+^/DsRed^+^ CiNs and the connecting DsRed single-positive neurons across the injection area in the cortex (*n* = 4 mice). **e** Illustrative figure of the His-EGFP^+^/DsRed^+^ CiNs and the connecting DsRed single-positive neuron. White arrowhead points to a CiN, and the asterisk highlights a local neuron showing direct synaptic connections with the CiN. **f** The three-dimensional reconstruction of the image in **e** (left). The arrowhead and asterisk point to the same cells in **e**. Scale bars, 100 μm; 20 μm in high-magnification (**b**); 40 μm (**e**). Error bars represent SEM.

We used retrograde mono-transsynaptic tracing^32^ to determine whether CiNs could form synaptic connection with host neurons. Two Cre-dependent flexed AAV vectors encoding avian tumor virus receptor A (TVA) with histone-EGFP (His-EGFP) and rabies virus glycoprotein (RVG) were injected into the cortex of *Gfap-cre* mice, restricting their expressions to astrocytes before reprogramming (Fig. 4a; Supplementary Fig. S10b). Then, at 7 wpi, we injected pseudotyped rabies virus carrying DsRed (RABV-DsRed) into the same site to label reprogrammed CiNs, which then showed as His-EGFP^+^/DsRed^+^. Consequently, host neurons forming synaptic connections with the CiNs were labeled as His-EGFP^–^/DsRed^+^ (Fig. 4a). When we tested the RABV tracing system in both the striatum and the cortex, we observed no DsRed background signal in the cortex but saw rare DsRed background signals in the striatum. Therefore, we conducted the RABV tracing experiment in the cortex.

Before the RABV tracing experiment, we had confirmed that our RABV-DsRed titer ensured specific infection of TVA-expressing neurons, but not other TVA-expressing non-neuronal cells. Rabies virus is a neurotropic virus that, at a low titer, mainly infects neurons in the brain^32–34^. To confirm the neurotropism of the RABV infection, we injected wild-type mice with AAV-CAG-Cre, AAV-EF1a-DIO-HIS-EGFP-2a-TVA, and AAV-EF1a-DIO-RVG, followed by RABV-DsRed. All the EGFP^+^/DsRed^+^ cells were NEUN positive, while none of NEUN^–^/EGFP^+^ cells were DsRed^+^ (Supplementary Fig. S10a), indicating that all cell types could be infected with AAV-EF1a-DIO-HIS-EGFP-2a-TVA, but only neurons could be infected by RABV. This result was consistent with our observation that DsRed^+^/His-EGFP^+^ cells were not found in the vehicle group (Fig. 4b; Supplementary Fig. S10c).

At 8 wpi, we observed His-EGFP^+^/DsRed^+^ CiNs, surrounded by His-EGFP^–^/DsRed^+^ cells in the same region of the cortex (Fig. 4b, c). The presence of both His-EGFP^+^/DsRed^+^ and His-EGFP^–^/DsRed^+^ cells in the chemically treated group indicated that the CiNs were innervated by host neurons (Fig. 4d–f). Collectively, the CiNs generated in situ showed the ability to receive synaptic projections from the host neurons.

## Discussion

In this study, we successfully developed a chemical approach to convert endogenous astrocytes into neurons in the adult mouse brain. Our CiNs displayed brain region-specific neuronal properties and mature electrophysiological characteristics. Additionally, CiNs showed the potential to connect with endogenous neurons, forming direct afferent connectivity with surrounding neurons in the cortex. These important characteristics of the CiNs suggest in vivo chemical reprogramming as a potential approach to regenerate functional compensatory neurons in the brain.

Using conditional lineage-tracing system, we demonstrated the resident astrocyte origin of CiNs. We carefully injected AAV-FLEX-EGFP into the striatum or cortex region of adult *Aldh1l1-cre* transgenic mice. It is important that the regulatory element of *Aldh1l1* was established by bacterial artificial chromosome (BAC)-based approach. Compared with AAV-based approach (AAV-hGFAP promoter), the ~228 kb C57BL/6J mouse BAC RP23-7M9 contained the entire *Aldh1l1* locus, which faithfully recapitulated the expression pattern of *Aldh1l1* gene. This system enabled us to achieve specific astrocyte labeling (Supplementary Fig. S6). Furthermore, by using a low titer and volume of injected AAV, we achieved astrocyte labeling restricted in the striatum or cortex, thus excluded the possibility that CiNs are derived from glial gene-expressing neural stem cells in the adult neurogenic zone (Supplementary Fig. S6a). Meanwhile, by initiating astrocyte labeling at adulthood, we excluded the mislabeling of endogenous neurons that transiently expressed astroglial genes during development (Supplementary Fig. S6).

In addition, we demonstrated that endogenous neurons were unlikely mislabeled during chemical reprogramming process. Firstly, we detected no GFAP or ALDH1L1 expression in AAV-hSyn-EGFP-labeled endogenous neurons at 1, 2, 4, and 8 wpi of the reprogramming process (Supplementary Fig. S8). Secondly, we genetically traced the transient astrocyte gene expression in resident neurons by injecting AAV-FLEX-EGFP into the striatum of *Aldh1l1-cre* mice. Meanwhile, we injected a specific retrograde neuronal tracer, Alexa 555-conjugated CTB into the ventral pallidum to retrogradely label the soma of striatal neurons. We did not observe EGFP^+^/CTB^+^ neurons at the core of the reprogramming area at 2 wpi, which demonstrated that astroglial gene was not activated in endogenous neurons during the chemical infusion period (Supplementary Fig. S9a, b). At 8 wpi, we did not observe EGFP^+^/CTB^+^ neurons either. However, since the cell excretion of CTB by the time of 8-wpi, the labeling of endogenous neurons were not as saturated as that of 2-wpi, our results could just suggest that astrocyte gene was unlikely activated in endogenous neurons during the whole reprogramming process (Supplementary Fig. S9b). Thirdly, we treated primary neuronal cultures isolated from *Gfap-cre/Rosa26-tdTomato/TauEGFP* mice with small molecules and detected no tdTomato expression at 16-dpi, indicating small molecule cocktail could not elicit astroglial gene expression in primary neurons in vitro (Supplementary Fig. S9c). Most importantly, we demonstrated that in vivo chemical reprogramming depended on a synergetic effect of small molecules. The cocktail without either Forskolin, I-BET151, ISX9, or CHIR99021 failed to generate CiNs both in vitro and in vivo (Supplementary Figs. S1d, S4b). This result further demonstrated that the generation of CiNs was induced by the synergetic function of each small molecules, instead of endogenous mislabeling. Collectively, these results demonstrated that CiNs were fate-mapped to an astrocytic origin in non-neurogenic regions.

In this study, the gradual astrocyte-to-neuron conversion was supported by the transcriptomic dynamics both in vitro and in vivo (Fig. 3; Supplementary Fig. S3). After chemical induction, CiNs exhibited neuronal morphology, expressed mature neuronal markers, and obtained functional electrophysiological properties (continuous APs, fast inward currents) (Figs. 1, 2; Supplementary Fig. S7). The connectivity of CiNs with surrounding neurons was confirmed by monosynaptic tracing using retrograde pseudotyped rabies virus (Fig. 4).

Interestingly, we found CiNs in situ resembled brain region-specific neuronal properties. In the striatum, CiNs mainly resembled some characteristics of GABAergic neurons. Some CiNs also expressed hallmarkers of striatum-specific neuronal subtypes, including DARPP32^+^ medium spiny neurons, and PVALB^+^ and NPY^+^ interneurons^35^ (Fig. 2b, c). In addition, through single-cell RNA sequencing analysis, we detected striatum-specific subtype hallmark genes (*Gad1*, *Ppp1r1b*, *Npy*, and *Pvalb*) in CiNs (Fig. 3g). In the cortex, we showed that chemical reprogramming mostly generated CiNs with glutamatergic neuronal characteristics, along with other cortex-specific neuronal markers, including CTIP2, TBR1, and PVALB, which were also detected in the CiNs^36^ (Fig. 2b, d). The CiNs’ subtype-specific characteristics resembled the endogenous neurons found in the same region of the brain, suggesting that the effect of local niche on coordinating the subtype of the newly generated CiNs. Interestingly, this observation of the effect of a local environmental niche on CiN neuronal subtype patterning was echoed by previous studies conducted on the native environmental impact on Ngn2-meditated reprogramming^37^. In their study, it showed that NGN2 overexpression generated GABAergic neurons in the striatum while generating glutamatergic neurons in the neocortex. In addition, Hu et al.^38^ reported that astrocytes derived from the cortex, cerebellum, and spinal cord exhibited biological heterogeneity and possessed distinct susceptibility to transcription factor-induced neuronal reprogramming. Based on those studies and our results, we hypothesized that the intrinsic properties of astrocytes from different brain regions and the local environment niche together contribute to the generation of different neuronal subtypes.

In summary, our proof-of-concept study demonstrated in vivo chemical reprogramming of astrocytes into functional neurons, suggesting a new path to regenerate neurons for brain repair^16–20^. To enhance the application potential of chemical-induced in vivo reprogramming, further improvement of drug delivery strategy would be needed. Controlled drug release techniques, including microsome entrapment, nanocarriers, and nanocrystals^39–41^, may allow us to achieve sustained drug release with a single injection, thus avoiding injury to the brain parenchyma. Importantly, an effective drug delivery strategy may also boost reprogramming efficiency. In addition, reducing the off-target effect of small molecules would further enhance the safety of chemically induced reprogramming for in vivo applications. Prodrug, which is used to improve the selectivity and reduce the off-target effect of a drug, may be a promising solution. Recently, we used the prodrug strategy to enhance the targeting on senescent cells and improve physical function in old age^42^. Future studies focusing on these two directions would enhance the functional repair potential of in vivo chemical reprogramming.

## Materials and methods

### Mice

Homozygous TauEGFP reporter mice (Stock No. 004779), *Gfap-cre* transgenic mice (Stock No. 024098), *Rosa-CAG-LSL-tdTomato* mice (Stock No. 007905) were purchased from the Jackson Laboratory. *Aldh1l1-cre* mice were obtained from the Mutant Mouse Regional Resource Center and were donated by the National Institute of Neurological Disorders and Stroke-funded GENSAT (Gene Expression Nervous System Atlas). TauEGFP heterozygous mice were generated by crossing the homozygous TauEGFP knock-in mice with wild-type ICR mice. *Gfap-cre/Rosa26-tdTomato* mice were generated by crossing the *Gfap-cre* transgenic mouse strain, which expresses Cre recombinase under the control of the glial fibrillary acidic protein (*GFAP*) promoter, with the *Rosa26-tdTomato* mouse strain, in which the Rosa26 locus was inserted using a CAG promoter and was followed by a LoxP-Stop-LoxP cassette-controlled fluorescent tdTomato locus. All animal experiments had received prior approval by the Animal Protection Guidelines of Peking University, China.

### Chemical infusion into the mouse brain

Small molecules were resolved in the artificial cerebrospinal fluid (aCSF) with 300 ng/mL bFGF as follows: Forskolin, 300 μM; CHIR, 60 μM; ISX9, 120 μM; I-BET151, 6 μM; Y27632, 30 μM. For chemical infusion, a total volume of 100 μL aCSF was loaded into the Alzet osmotic minipumps to obtain a constant release at the rate of 0.25 μL/h for 14 days (Alzet 1002; Cupertino, CA). In the vehicle group, same amount of aCSF without small molecules was infused into the mouse brains. Stereotaxic surgeries were performed on two-month-old mice for chemical infusion. Mice were anesthetized with 0.8% Pelltobarbitalum Natricum and heads were shaved and swabbed with 70% ethanol and iodophor. Erythromycin eye ointment was applied to prevent corneal drying and a heat pad (RWD, Shenzhen, China) was used to hold body temperature at 37 °C. A small midline incision was made to expose the skull. Periosteal connective tissues adhering to the skull were removed. The skull was kept dry permitting good adhesion of the dental cement which was later used to secure the cannula. The L-shaped cannula was inserted into the striatum (coordinates: AP 0.5 mm, ML –2 mm, DV –3 mm) or the cortex (coordinates: AP 0.5 mm, ML –2 mm, DV –0.5 mm) and the pump subcutaneously on the back. The cannula was secured to the skull with instant adhesive (Tonsan 1454, China) and dental cement. The incision was closed by suture thread. Animals were then placed in a clean warm cage on a heating pad until mobile.

### Virus injection

For virus injection, the micropipette was held in place before the injection for 5 min. Viruses were then injected into the cortex at a constant rate of 0.1 μL per min and at 0.02 μL per min into the striatum. To prevent any backflow, the pipette was held for another 5 min before the slowly withdrawing. All viruses used in this study were purchased from BrainVTA Wuhan, China.

For chemical induction in conditional lineage-tracing systems, to trace the astrocytes in *Aldh1l1-cre* and *Gfap-cre* mice, a total volume of 0.1 μL of rAAV-CAG-Dio-EGFP-WPRE-pA (0.94 × 10^12^ vg/mL) was injected into the striatum (coordinates: AP 0.5 mm; ML –2 mm; DV –3 mm), or 0.25 μL for cortex (coordinates: AP 0.5 mm; ML –2 mm; DV –0.5 mm). One week later, osmatic pump infused with small molecules was implanted into the mouse brain at the same coordinates. To label GABAergic neurons in the striatum, a total volume of 0.3 μL of rAAV-vGAT-mCherry-WPRE-pA (0.93 × 10^12^ vg/mL) was injected into the same coordinates of the striatum after chemical treatment (≥ 8 wpi). To label glutamatergic neurons in the cortex, a total volume of 0.3 μL of rAAV-vGLUT2-mCherry-WPRE-hGH (1.00 × 10^12^ vg/mL) was injected into the same coordinates of the cortex after chemical treatment (≥ 8 wpi).

To label resident neurons, a total volume of 0.5 μL of rAAV-hSyn-EGFP-WPRE-pA (2.09 × 10^12^ vg/mL) was injected into the striatum and the cortex.

For CTB labeling, a total volume of 0.5 μL of 0.5% CTB was injected into the ventral pallidum (coordinates: AP 1.5 mm; ML 0 mm; DV –4.2 mm).

For RABV tracing experiments, to trace the astrocytes, *Gfap-cre* mice were used, and a total volume of 0.5 μL mixed with rAAV-EF1α-DIO-His-EGFP-2a-TVA-WPRE-pA (3.7 × 10^12^ vg/mL) and rAAV-EF1α-DIO-RVG-WPRE-pA (3.6 × 10^12^ vg/mL) at the ratio of 1:1 was injected into the cortex (coordinates: AP 0.5 mm; ML –2 mm; DV –0.5 mm). One week later, similar procedures as described above for chemical treatment were adopted, which was within the cortex and the same coordinates of the viral injection. To label the induced neurons, 0.3 μL of RABV-ENVA-ΔG-DsRed (1.0 × 10^8^ IFU/mL) was injected into the same site one week prior to perfusion. Brain slices were made as 30 μm thick.

To test the specificity of RABV virus labeling, wild-type mice were used and 0.5 μL mixture of AAV-CAG-Cre (1.0 × 10^12^ vg/mL), AAV-EF1a-DIO-HIS-EGFP-2a-TVA (1.2 × 10^12^ vg/mL), and AAV-EF1a-DIO-RVG was injected into the cortex (coordinates: AP 0.5 mm; ML –2 mm; DV –0.5 mm), followed by RABV-DsRed injection a week later.

Additional information has been described in the supplementary information.

## Supplementary information

Supplementary information
