# Supplementary material for: In vivo chemical reprogramming of astrocytes into neurons
Source: Cell Discov. 2021 Mar 2;7:12. doi: 10.1038/s41421-021-00243-8 (PMC7921425; doi:10.1038/s41421-021-00243-8)
Supplement: Supplementary file 1 — Supplementary information [file 41421_2021_243_MOESM1_ESM.pdf]

Supplementary Information

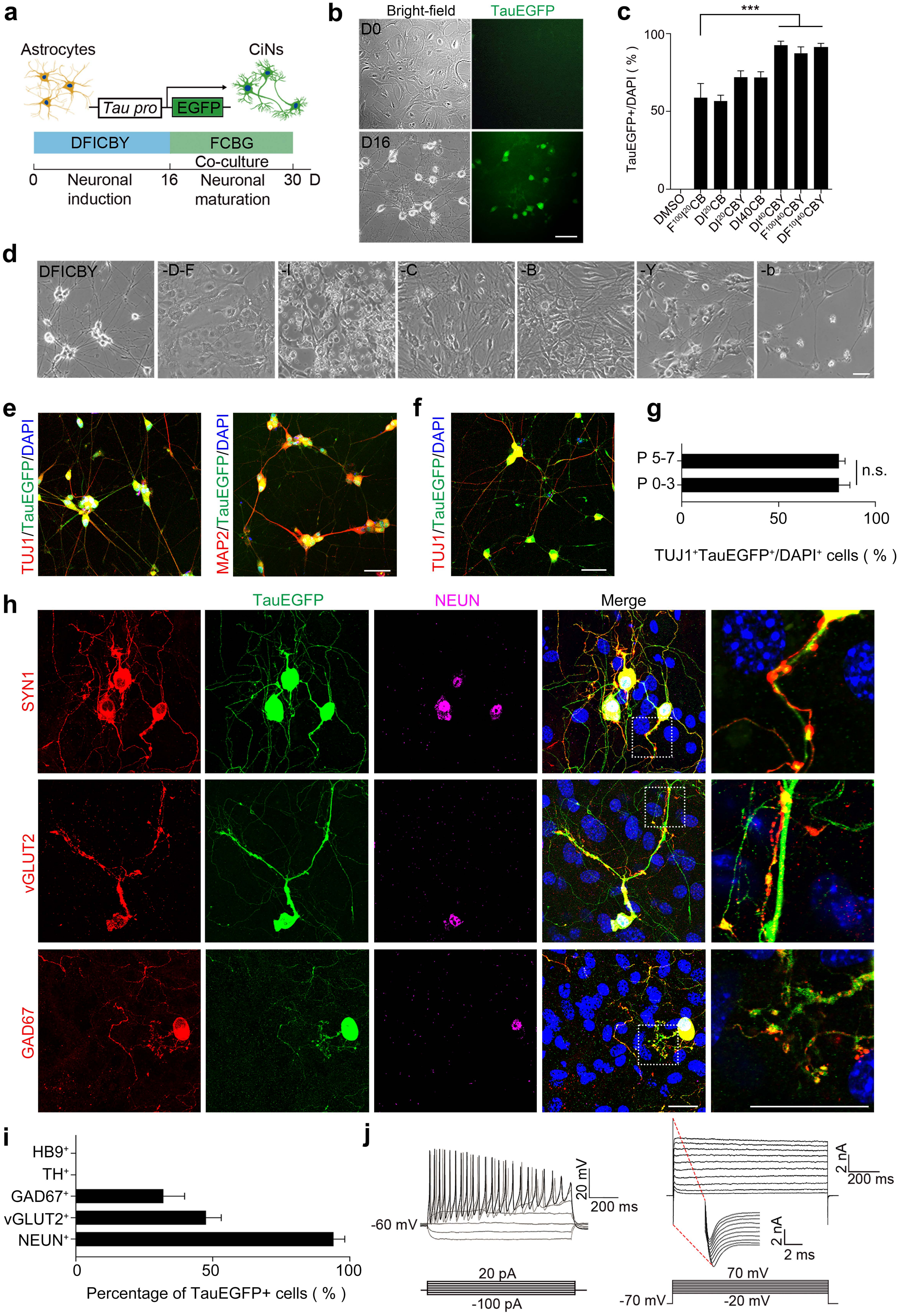

a
Astrocytes
CiNs
Tau pro
EGFP
DFICBY
FCBG
Co-culture
0
16
30
D
Neuronal induction
Neuronal maturation
b
Bright-field
TauEGFP
D0
D16
c
***
TauEGFP+/DAPI (%)
100
50
0
DMSO
d
DFICBY
-D-F
-I
-C
-B
-Y
-b
e
TUJ1/TauEGFP/DAPI
MAP2/TauEGFP/DAPI
f
TUJ1/TauEGFP/DAPI
g
P 5-7
P 0-3
n.s.
0
50
100
TUJ1+TauEGFP+/DAPI+ cells (%)
h
TauEGFP
NEUN
Merge
SYN1
vGLUT2
GAD67
i
HB9+
TH+
GAD67+
vGLUT2+
NEUN+
0
50
100
Percentage of TauEGFP+ cells (%)
j
20 mV
200 ms
-60 mV
20 pA
-100 pA
2 nA
200 ms
2 nA
2 ms
70 mV
-70 mV
-20 mV

Supplementary Fig. S1. A chemical cocktail efficiently converts astrocytes into functional neuronal cells in vitro.

a, Schematic diagram of chemically induced astrocyte-to-neuron reprogramming. **b**, Representative images of $TauEGFP^-$ astrocytes (D0) and $TauEGFP^+$ cells induced by FICB at 16-dpi. **c**, Percentages of $TauEGFP^+$ cells among total cells at 16-dpi after chemical treatment (n = 5 biological repeats). D, DBcAMP, 100 μM; F, Forskolin, 100/10 μM; I, ISX9, 20/40 μM; C, CHIR99021, 20 μM; B, I-BET151, 2μM and Y, Y-27632, 10 μM. An optimized cocktail DFICBY (DBcAMP, Forskolin, ISX9, CHIR99021, I-BET151 and Y-27632) enhanced neuronal conversion efficiency, yielding 91.1 ± 2.6 % of $TauEGFP^+$ cells. ***p < 0.001 by one-way ANOVA with Tukey's Multiple Comparison Test. **d**, Representative bright field images of cells treated by single molecule omission from DFICBY cocktail at 16-dpi. **e**, Immunofluorescence analyses of $TauEGFP^+$ cells at 16-dpi for pan-neuronal markers (TUJ1 and MAP2). **f**, $TauEGFP^+$ cells induced from astrocytes isolated during postnatal days-5–7 expressed TUJ1 at 16-dpi. **g**, Conversion efficiency comparison of astrocytes isolated during postnatal days-0–3 and postnatal days-5–7. n.s. by unpaired t test (n = 3 biological repeats). **h**, $TauEGFP^+$ cells at 30-dpi expressed mature neuron marker NEUN, glutamatergic neuron marker VGLUT2 and GABAergic neuron marker GAD67 after co-culture with primary astrocytes. **i**, Quantification of $VGLUT2^+$, $GAD67^+$, TH^+ and $HB9^+$ cells (n = 3 biological repeats). **j**, Electrophysiological analyses of the induced $TauEGFP^+$ CiNs; action potentials and inactivating inward currents were recorded at 30-dpi.

Scale bars: 50 μm (b, d, e and f); 25 μm (h). Error bars represent s. e. m.

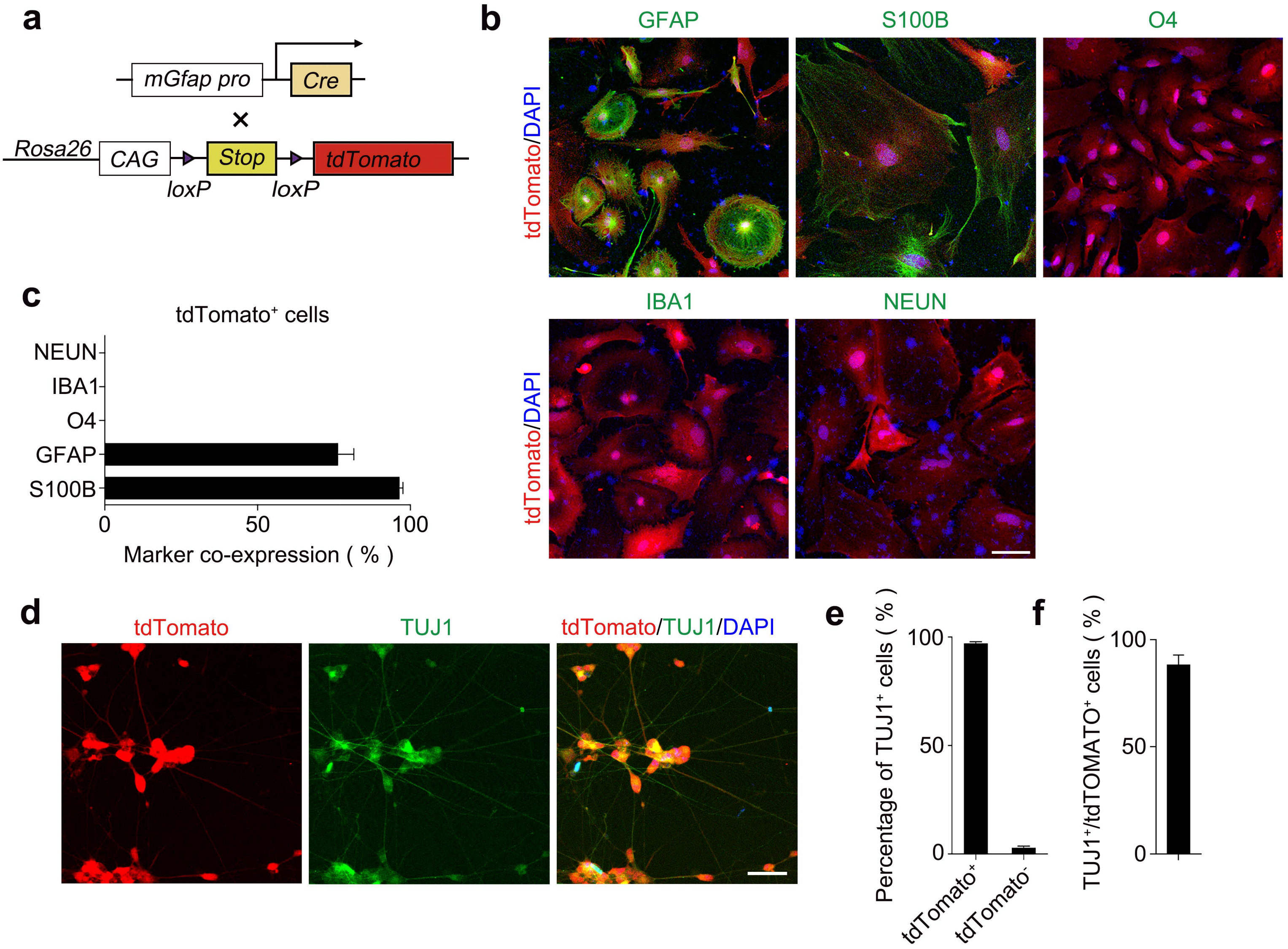
a
mGfap pro
Cre
×
Rosa26
CAG
Stop
tdTomato
loxP
loxP
b
GFAP
S100B
O4
IBA1
NEUN
tdTomato/DAPI
tdTomato/DAPI
c
tdTomato⁺ cells
NEUN
IBA1
O4
GFAP
S100B
0
50
100
Marker co-expression (%)
d
tdTomato
TUJ1
tdTomato/TUJ1/DAPI
e
Percentage of TUJ1⁺ cells (%)
100
50
0
tdTomato⁺
tdTomato⁻
f
TUJ1⁺/tdTOMATO⁺ cells (%)
100
50
0

Supplementary Fig. S2. Tracing of CiNs in Gfap-cre/Rosa26-tdTomato system in vitro.

a, Scheme of lineage-tracing constructs. **b**, Immunofluorescence of $tdTomato^+$ cells for astrocytes markers GFAP and S100B, oligodendrocytes marker O4, microglia marker IBA1 and neuronal marker NEUN. **c**, Characterization of $tdTomato^+$ cells. 96.5 ± 1.1 % $tdTomato^+$ cells were $S100B^+$, 76.4 ± 5.1 % $tdTomato^+$ cells were $GFAP^+$. All the $tdTomato^+$ cells were negative for neuronal marker NEUN, microglial marker IBA1and oligodendrocyte marker O4 (*n* = 3 biological repeats). **d**, Immunofluorescence analyses of $tdTomato^+$ cells at 16-dpi for pan-neuronal marker TUJ1. **e**, Quantification, in percentage, of $tdTomato^+/TUJ1^+$ cells induced from astrocytes labeled by tdTomato at 16-dpi (*n* = 5 biological repeats). **f**, Conversion efficiency quantified by the percentage of $TUJ1^+$ cells in all $tdTomato^+$ cells at 16-dpi. (*n* = 3 biological repeats).

Scale bars: 100 μm (b); 50 μm (d). Error bars represent s. e. m.

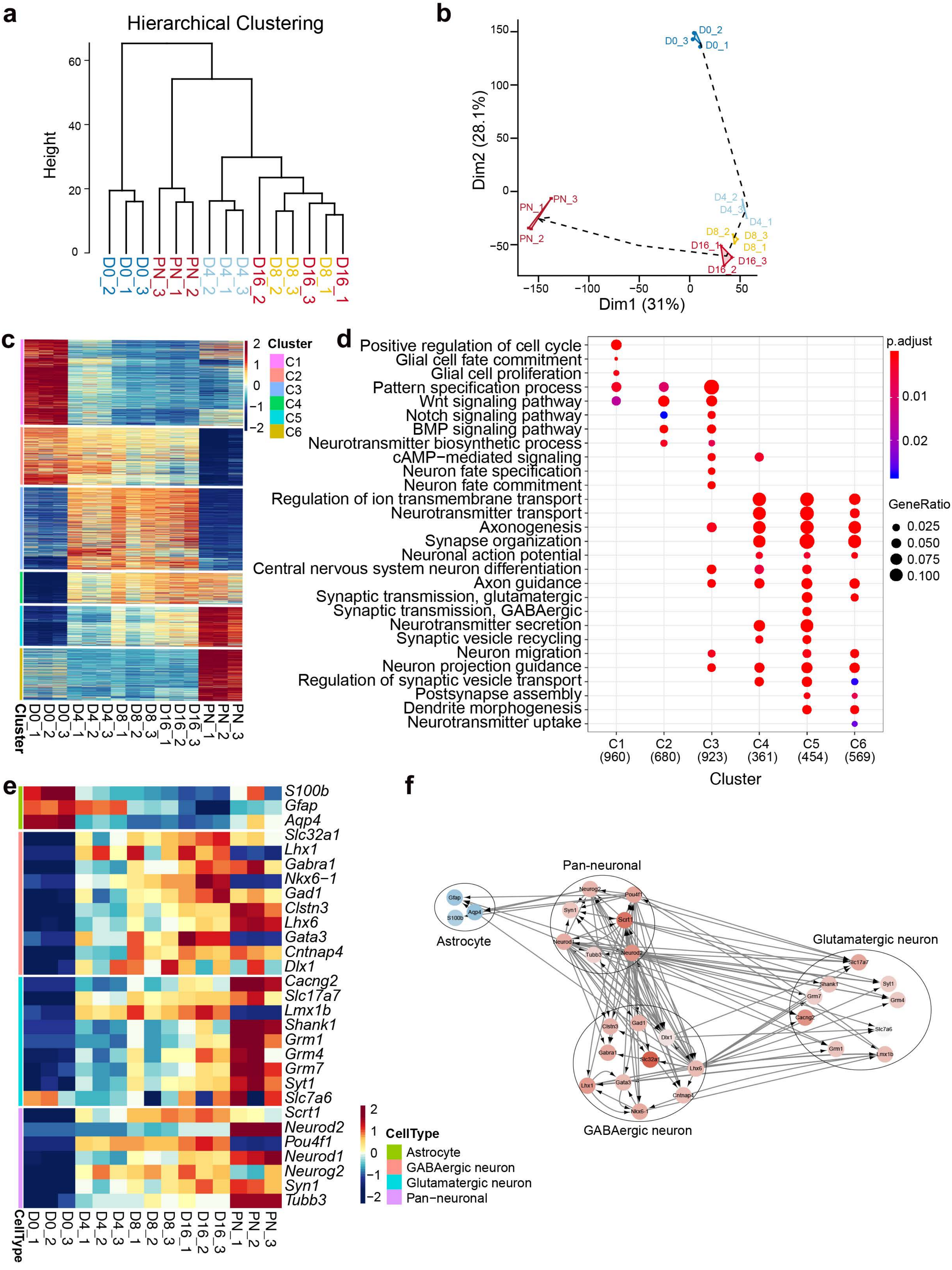
a
Hierarchical Clustering
Height
D0_2 D0_1 D0_3 PN_3 PN_1 PN_2 D4_2 D4_1 D4_3 D16_2 D8_2 D8_3 D16_3 D8_1 D16_1
b
Dim2 (28.1%)
Dim1 (31%)
c
Cluster
C1 C2 C3 C4 C5 C6
d
Positive regulation of cell cycle
Glial cell fate commitment
Glial cell proliferation
Pattern specification process
Wnt signaling pathway
Notch signaling pathway
BMP signaling pathway
Neurotransmitter biosynthetic process
cAMP−mediated signaling
Neuron fate specification
Neuron fate commitment
Regulation of ion transmembrane transport
Neurotransmitter transport
Axonogenesis
Synapse organization
Neuronal action potential
Central nervous system neuron differentiation
Axon guidance
Synaptic transmission, glutamatergic
Synaptic transmission, GABAergic
Neurotransmitter secretion
Synaptic vesicle recycling
Neuron migration
Neuron projection guidance
Regulation of synaptic vesicle transport
Postsynapse assembly
Dendrite morphogenesis
Neurotransmitter uptake
C1 (960) C2 (680) C3 (923) C4 (361) C5 (454) C6 (569)
Cluster
p.adjust
GeneRatio
e
S100b Gfap Aqp4 Slc32a1 Lhx1 Gabra1 Nkx6−1 Gad1 Clstn3 Lhx6 Gata3 Cntnap4 Dlx1 Cacng2 Slc17a7 Lmx1b Shank1 Grm1 Grm4 Grm7 Syt1 Slc7a6 Scrt1 Neurod2 Pou4f1 Neurod1 Neurog2 Syn1 Tubb3
CellType
Astrocyte
GABAergic neuron
Glutamatergic neuron
Pan−neuronal
f
Astrocyte
Pan-neuronal
Glutamatergic neuron
GABAergic neuron

Supplementary Fig. S3. RNA-sequencing analysis of transcriptome dynamics of chemical-induced reprogramming in vitro.

a, Hierarchical cluster analysis of samples at different time points during reprogramming and primary neuron. To compare gene expression profile among reprogramming neurons with primary neurons, differential genes among D0 and primary neurons (PN) with which absolute value of leading log-fold-change greater than 4 and FDR < 0.01 were used to build hierarchical clustering trees. **b**, Principal component analysis of samples at different time points during reprogramming and primary neurons. (PN, primary neuron). **c**, Heatmap of differentially expressed genes during reprogramming process in vitro. Genes with absolute value of leading log-fold-change greater than 2 and FDR < 0.01 were defined as differential expressed genes. **d**, GO analysis of differentially expressed genes in each sample. **e**, Heatmap presenting down-regulated astrocyte hallmark genes and up-regulated transcription factors and hallmark genes of pan-neurons, glutamatergic neurons and GABAergic neurons. **f**, Gene regulation network analysis of typical down-regulated and up-regulated genes during reprogramming process.

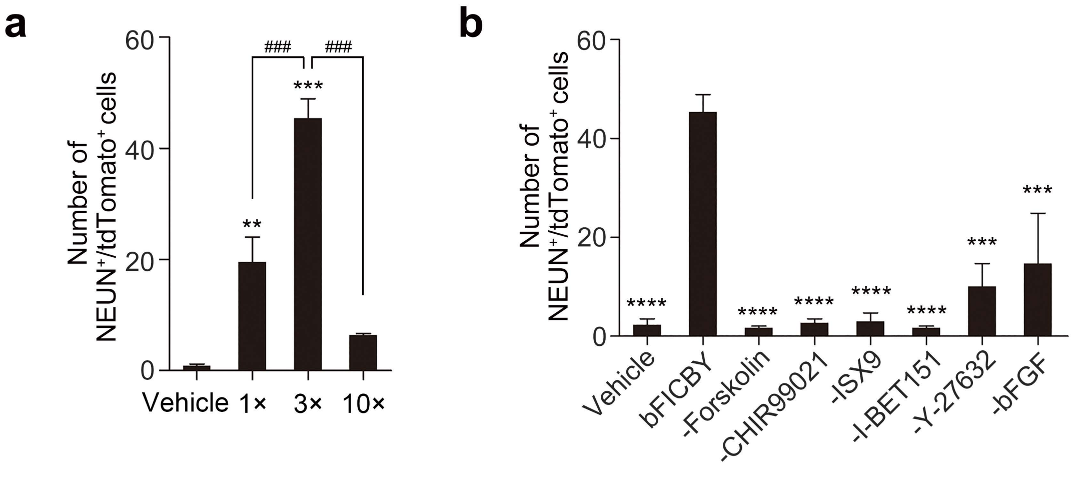

a
b
Number of NEUN+/tdTomato+ cells
60
40
20
0
###
###
***
**
Vehicle
1×
3×
10×
****
****
****
****
****
***
***
Vehicle
bFICBY
-Forskolin
-CHIR99021
-ISX9
-I-BET151
-Y-27632
-bFGF

Supplementary Fig. S4. Optimization and confirmation of chemical cocktails for in vivo reprogramming.

a, Dosage optimization of the bFICBY cocktail in adult Gfap-cre/Rosa26-tdTomato mouse striatum. Quantification of $tdTomato^+/NEUN^+$ cells per slice in chemical treated mice at 1, 3, and 10 folds of dosage used in vitro (vehicle group, *n* = 5 mice; 1×, one-fold dosage group, *n* = 4 mice; 3×, triple dosage group, *n* = 3 mice; 10×, 10-fold dosage group, *n* = 3 mice). **b**, Quantification of $tdTomato^+/NEUN^+$ cells in single-small-molecule-omission groups at 8-wpi, compared with bFICBY group.

Error bars represent s. e. m. $^{**}p < 0.01$; $^{***}p < 0.001$; $^{****}p < 0.0001$. Comparisons of dosage groups (a) are indicated in the same manner but using the symbol "#".

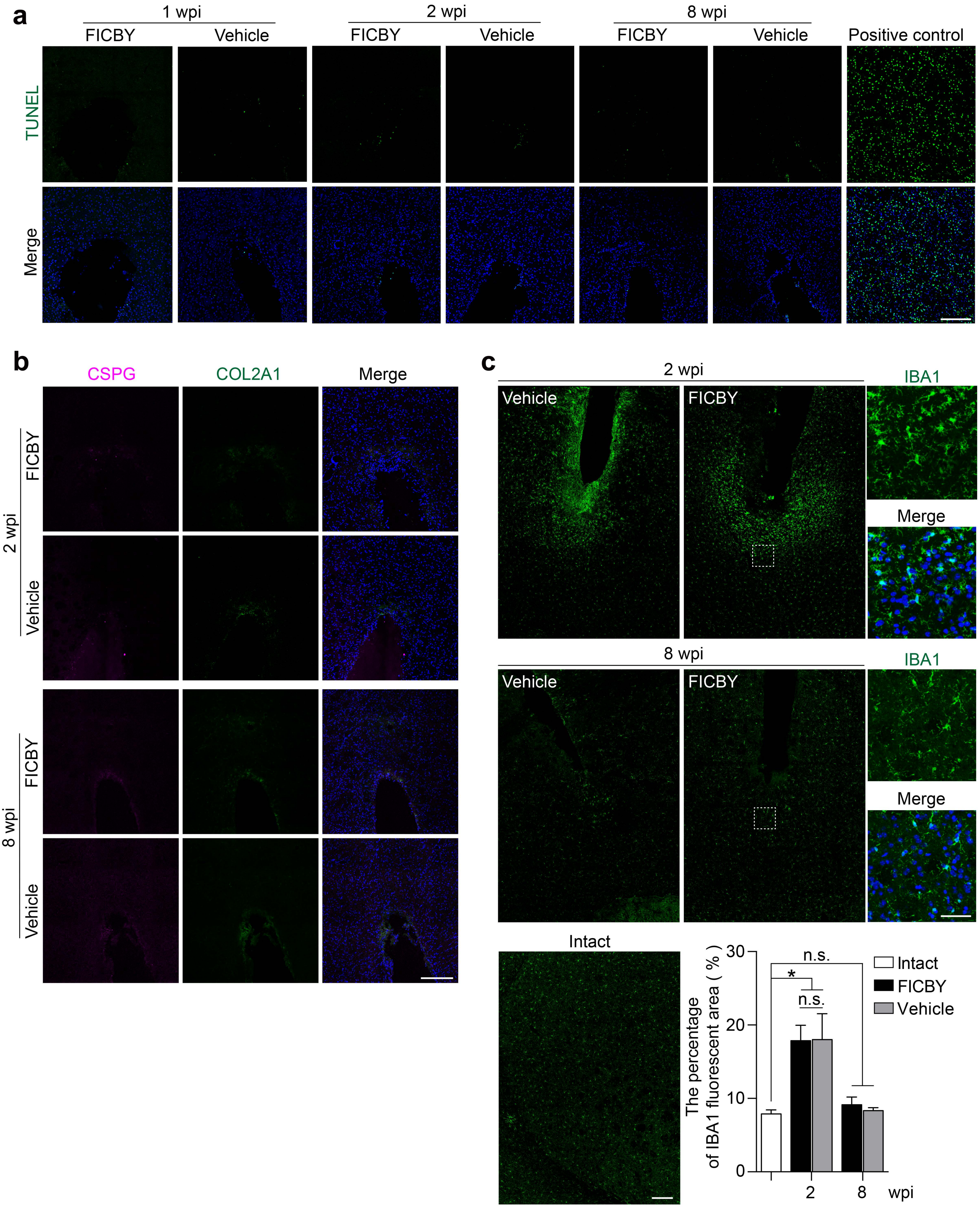
a
1 wpi
2 wpi
8 wpi
FICBY
Vehicle
Positive control
TUNEL
Merge
b
CSPG
COL2A1
Merge
2 wpi
8 wpi
FICBY
Vehicle
c
2 wpi
8 wpi
Vehicle
FICBY
IBA1
Merge
Intact
The percentage of IBA1 fluorescent area (%)
n.s.
*
Intact
FICBY
Vehicle
0
10
20
30
2
8
wpi

Supplementary Fig. S5. Small molecules could not cause cell apoptosis, glial scar, or sustained inflammation.

a, Immunofluorescence analyses of $TUNEL^+$ cells at 1-, 2- and 8-wpi in the chemical treated groups, vehicle groups and positive control. (n = 3 mice). **b**, Immunofluorescence analyses of glial scar markers, CSPG and COL2A1 at 2- and 8-wpi in vehicle groups and chemical treated groups. (n = 3 mice). **c**, Immunofluorescence analyses and quantification of microglial markers IBA1 at 2- and 8-wpi in intact groups, vehicle groups and chemical treated groups. (n = 3 mice).

Scale bars: 200 μm (a, b); 200 μm, 50 μm in high-magnification panels (c). error bars represent s. e. m. *$p < 0.05$ by one-way ANOVA with Tukey's Multiple Comparison Test.

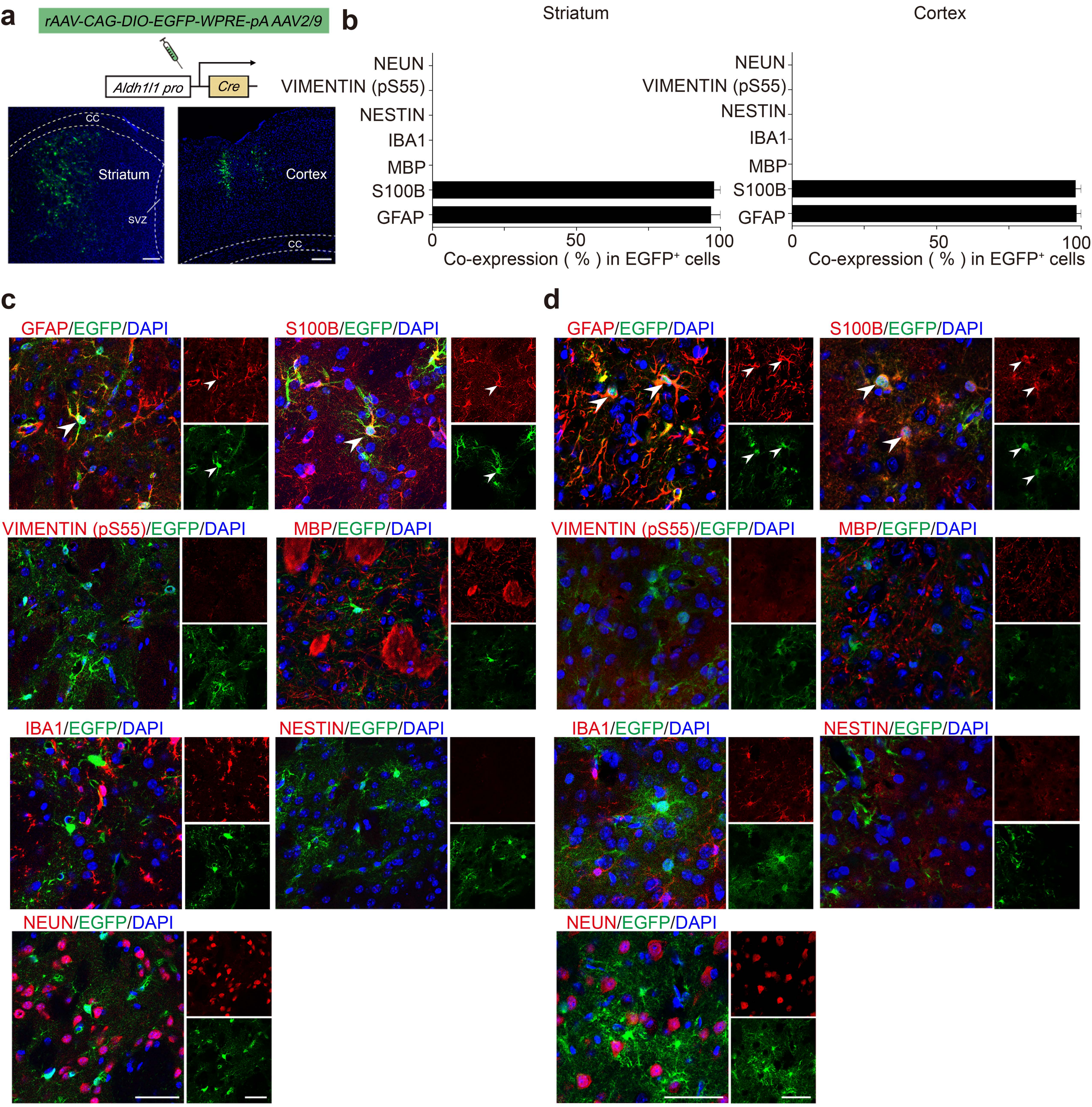
a
rAAV-CAG-DIO-EGFP-WPRE-pA AAV2/9
Aldh1l1 pro
Cre
cc
Striatum
svz
Cortex
cc
b
Striatum
Cortex
NEUN
VIMENTIN (pS55)
NESTIN
IBA1
MBP
S100B
GFAP
0
50
100
Co-expression (%) in EGFP+ cells
c
GFAP/EGFP/DAPI
S100B/EGFP/DAPI
VIMENTIN (pS55)/EGFP/DAPI
MBP/EGFP/DAPI
IBA1/EGFP/DAPI
NESTIN/EGFP/DAPI
NEUN/EGFP/DAPI
d
GFAP/EGFP/DAPI
S100B/EGFP/DAPI
VIMENTIN (pS55)/EGFP/DAPI
MBP/EGFP/DAPI
IBA1/EGFP/DAPI
NESTIN/EGFP/DAPI
NEUN/EGFP/DAPI

Supplementary Fig. S6. Astrocytes labelling specificity in Aldh1l1-cre mice, related to Fig. 1.

a, Scheme of conditional lineage tracing. AAV-FLEX-EGFP vector was injected in the striatum or the cortex of Aldh1l1-cre mice. Representative images showed the labelling area of AAV-FLEX-EGFP vector, which was restricted in striatum or cortex. **b**, Cell identity characterization of $EGFP^+$ cells in conditional lineage-tracing mice (*n* = 3 mice). **c**, Immunofluorescence of $EGFP^+$ cells in the striatum of conditional lineage-tracing mice. Arrowheads represent co-localization of $EGFP^+$ and corresponding markers. **d**, Immunofluorescence of $EGFP^+$ cells in the cortex of conditional lineage-tracing mice. Arrowheads represent co-localization of $EGFP^+$ and corresponding markers.

Scale bars: 200 μm (a), 50 μm (c and d). Error bars represent s. e. m.

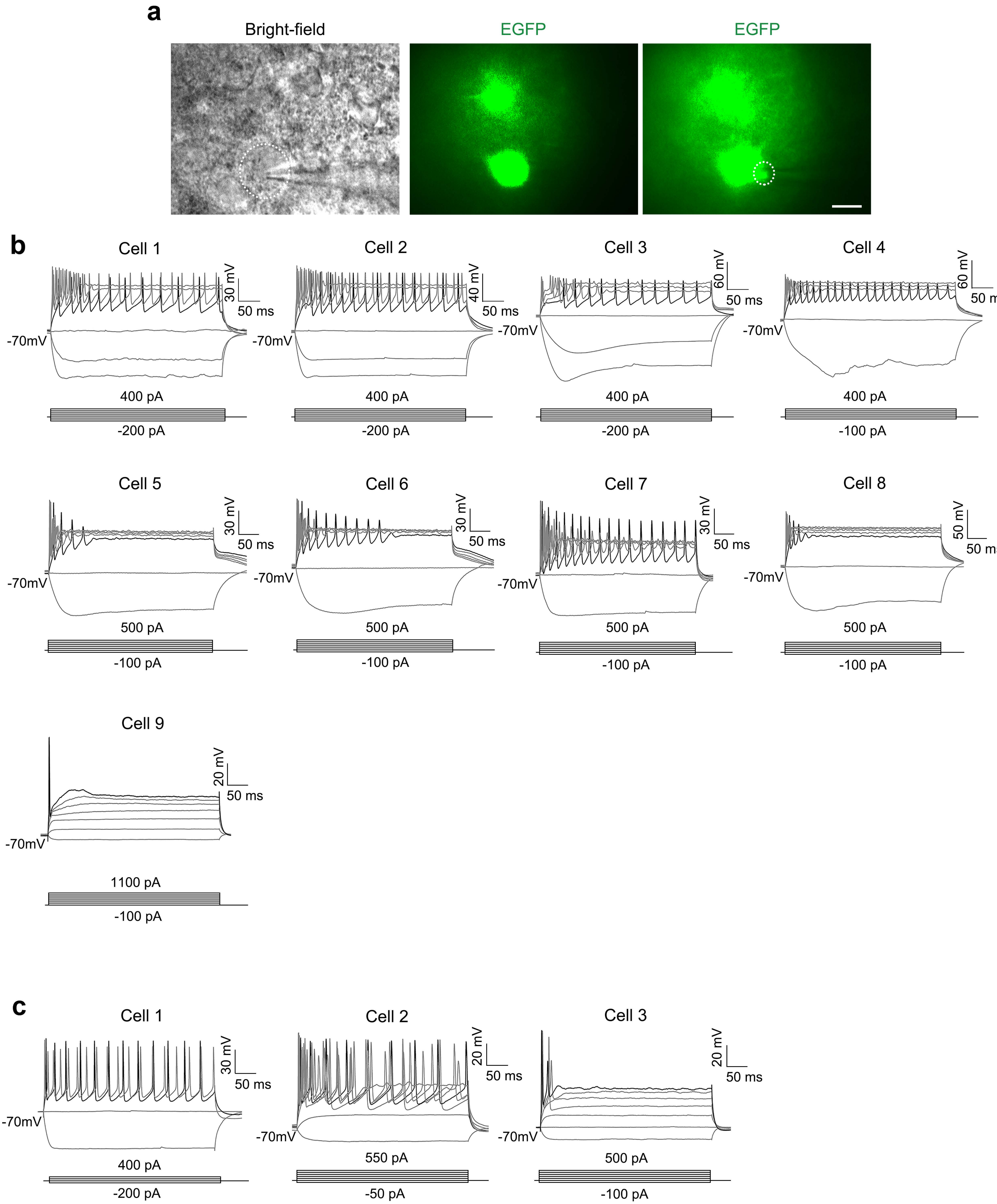

a
Bright-field
EGFP
EGFP
b
Cell 1
30 mV
50 ms
-70mV
400 pA
-200 pA
Cell 2
40 mV
50 ms
-70mV
400 pA
-200 pA
Cell 3
60 mV
50 ms
-70mV
400 pA
-200 pA
Cell 4
60 mV
50 ms
-70mV
400 pA
-100 pA
Cell 5
30 mV
50 ms
-70mV
500 pA
-100 pA
Cell 6
30 mV
50 ms
-70mV
500 pA
-100 pA
Cell 7
30 mV
50 ms
-70mV
500 pA
-100 pA
Cell 8
50 mV
50 ms
-70mV
500 pA
-100 pA
Cell 9
20 mV
50 ms
-70mV
1100 pA
-100 pA
c
Cell 1
30 mV
50 ms
-70mV
400 pA
-200 pA
Cell 2
20 mV
50 ms
-70mV
550 pA
-50 pA
Cell 3
20 mV
50 ms
-70mV
500 pA
-100 pA

Supplementary Fig. S7. Representative action potential firing patterns and electrophysiological parameters of the CiNs that induced in Aldh1l1-cre mice, related to Fig. 1.

a, Representative images of $EGFP^+$ cells in the brain slice for patch-clamp recordings. Right image showed the fluorescence within the pipette, which was outlined by the white broken circle. **b**, Action potentials are shown for all 9 cells recorded in the striatum of Aldh1l1-cre mice at 9–14-wpi. Data of Cell 1 is also presented in Fig. 1f as the example of all recorded cells. **c**, Action potentials are shown for all 3 cells recorded in the cortex of Aldh1l1-cre mice at 9–10-wpi. Data of Cell 1 is also presented in Fig. 1g as the example of all recorded cells.

Scale bar: 10 μm (a).

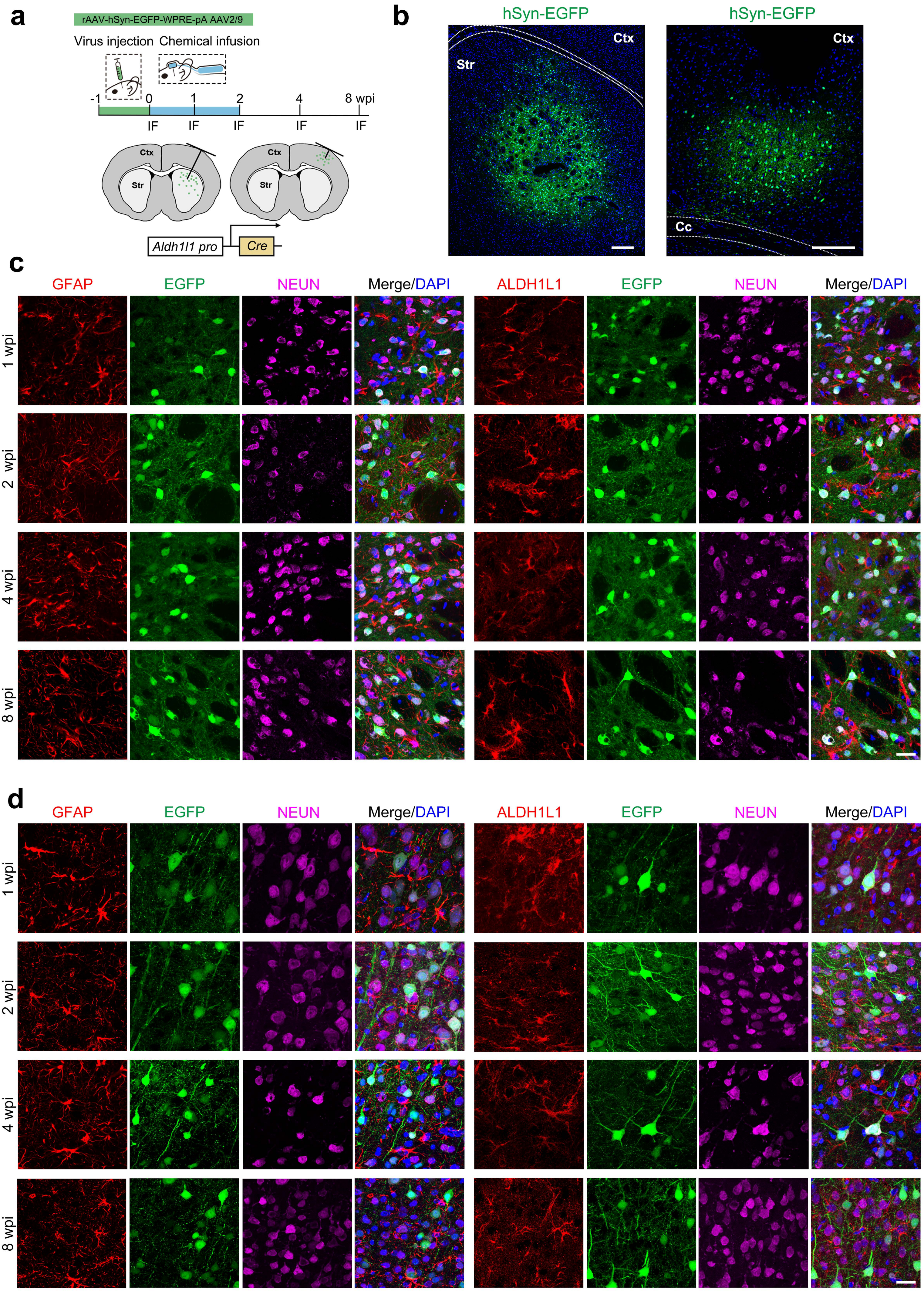
a
rAAV-hSyn-EGFP-WPRE-pA AAV2/9
Virus injection
Chemical infusion
-1
0
1
2
4
8 wpi
IF
IF
IF
IF
IF
Ctx
Str
Ctx
Str
Aldh1l1 pro
Cre
b
hSyn-EGFP
hSyn-EGFP
Ctx
Str
Ctx
Cc
c
GFAP
EGFP
NEUN
Merge/DAPI
ALDH1L1
EGFP
NEUN
Merge/DAPI
1 wpi
2 wpi
4 wpi
8 wpi
d
GFAP
EGFP
NEUN
Merge/DAPI
ALDH1L1
EGFP
NEUN
Merge/DAPI
1 wpi
2 wpi
4 wpi
8 wpi

Supplementary Fig. S8. Resident neurons did not express ALDH1L1 or GFAP during reprogramming process.

a, Scheme of resident neuron labeling by AAV-hSyn-EGFP. The chemical infusion was performed 1 week after AAV injection. **b**, Representative images showing the labelling area of resident neurons in striatum and cortex. **c**, Immunofluorescence analyses of $EGFP^+$/$NEUN^+$ resident neurons in the striatum for GFAP and ALDH1L1 at 1-, 2-, 4-, and 8-wpi. No ALDH1L1 or GFAP expression were detected in over 500 resident neurons (n = 3 mice). **d**, Immunofluorescence analyses of $EGFP^+$/$NEUN^+$ resident neurons in the cortex for GFAP and ALDH1L1 at 1-, 2-, 4-, and 8-wpi. No ALDH1L1 or GFAP expression were detected in over 500 resident neurons (n = 3 mice).

Scale bars: 100 μm (b); 25 μm (c and d)

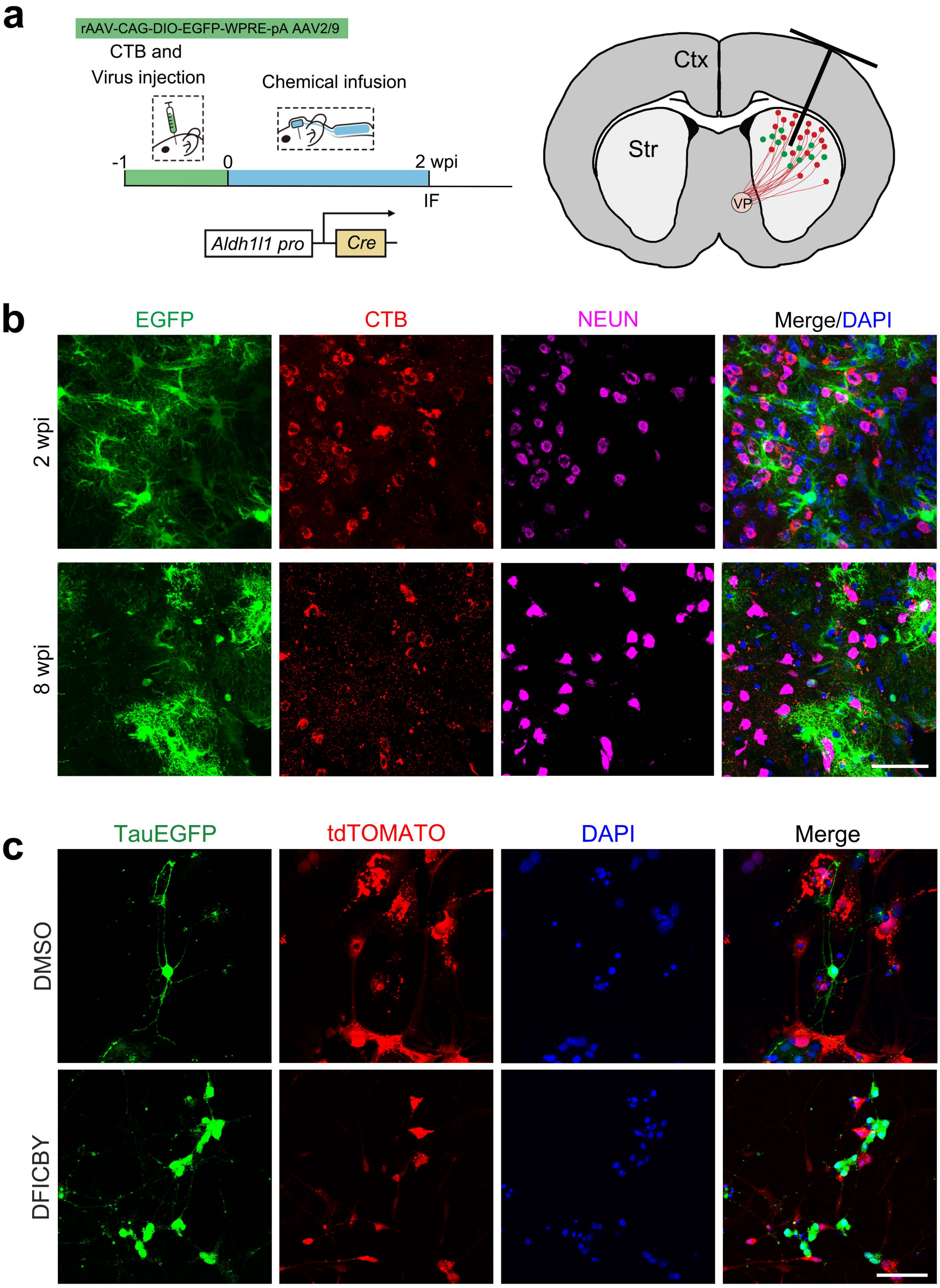

a
rAAV-CAG-DIO-EGFP-WPRE-pA AAV2/9
CTB and
Virus injection
Chemical infusion
-1
0
2 wpi
IF
Aldh1l1 pro
Cre
Ctx
Str
VP
b
EGFP
CTB
NEUN
Merge/DAPI
2 wpi
8 wpi
c
TauEGFP
tdTOMATO
DAPI
Merge
DMSO
DFICBY

Supplementary Fig. S9. Exclusion of transient astrocyte gene expressions in neurons. **a**, Scheme of striatal neuron labeling by Alexa Fluor 555 conjugated cholera toxin subunit B in Aldh1l1-cre mice injected with AAV-FLEX-EGFP. **b**, Representative images showing that CTB^+ resident neurons were not labeled by EGFP at both 2-wpi and 8-wpi (n = 3 mice). **c**, Co-culture of neurons isolated from Gfap-Cre/Rosa26-tdTomato/TauEGFP mice with astrocytes isolated from Gfap-Cre/Rosa26-tdTomato mice under DFICBY induction condition or DMSO. The results showed that neurons co-cultured with astrocytes would not be labeled by tdTomato upon treatment with DFICBY at 16-dpi.

Scale bars: 50 μm (b); 75 μm (c).

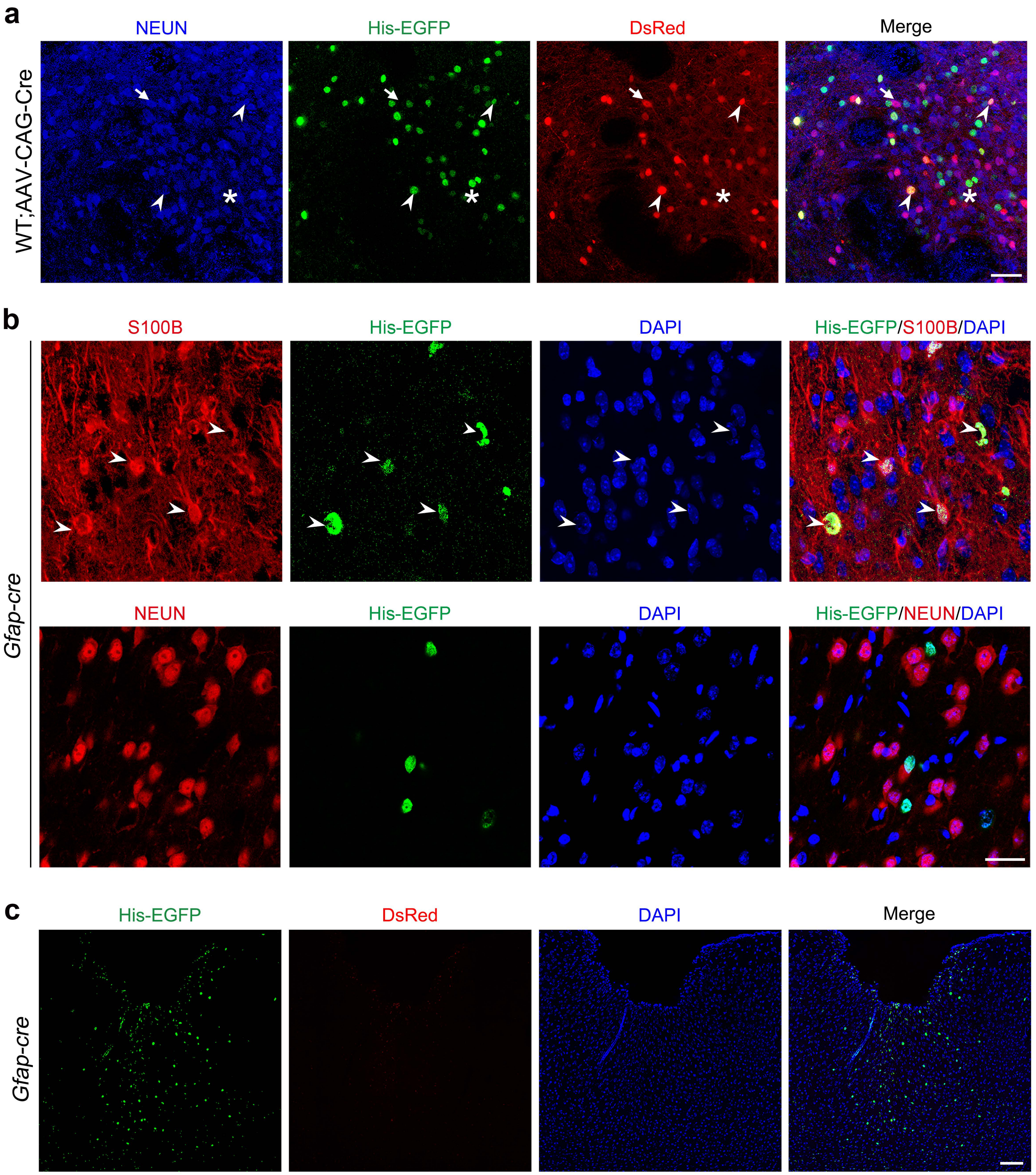
a
NEUN
His-EGFP
DsRed
Merge
WT;AAV-CAG-Cre
b
S100B
His-EGFP
DAPI
His-EGFP/S100B/DAPI
NEUN
His-EGFP
DAPI
His-EGFP/NEUN/DAPI
Gfap-cre
c
His-EGFP
DsRed
DAPI
Merge
Gfap-cre

Supplementary Fig. S10. Validation of the specificity of RABV tracing system.

a, NEUN immunostaining of the wild type mice injected with AAV-CAG-Cre, AAV-EF1a-DIO-HIS-EGFP-2a-TVA, and AAV-EF1a-DIO-RVG, followed by RABV-DsRed injection. Neurons and other cell types (asterisk) could all be infected with AAV-EF1a-DIO-HIS-EGFP-2a-TVA. But only resident neurons (arrowheads; $NEUN^{+}$/His-$EGFP^{+}$/$DsRed^{+}$) that already expressed TVA could be infected by RABV, which could transmit to the connected neuron (arrows; $NEUN^{+}$/$DsRed^{+}$). All of the $DsRed^{+}$ cells expressed NEUN, but none of $NEUN^{-}$/His-$EGFP^{+}$ cells were DsRed positive. **b**, Immunofluorescence analyses on Gfap-cre mice after infected with AAV-EF1a-DIO-HIS-EGFP-2a-TVA. Arrowheads highlight the co-expression of His-EGFP and S100B. Co-expression of His-EGFP and NEUN was not detected, indicating that helper viruses were expressed restrictedly in the cortical astrocytes. **c**, Confocal images of Gfap-cre mice injected with two helper virus, AAV-EF1a-DIO-HIS-EGFP-2a-TVA, and AAV-EF1a-DIO-RVG, followed by RABV-DsRed injection. RABV-DsRed could not infect astrocytes expressing TVA (His-$EGFP^{+}$).

Scale bars: 50 μm (a) 25 μm (b); 100 μm (c).

Supplementary Table S1. Electrophysiological properties of induced neurons after co-culture, related to Supplementary Fig. S1.

Induction	Cm (pF)	RMP (mV)	APthreshold (mV)	APamp (mV)	Maximal inward currents (pA)	N
CiNs	21.98 ± 2.64	-70.09 ± 2.08	-32.50 ± 1.54	42.92 ± 4.35	1249.0 ± 215.1	12/12
Cell1	16.06	-84.23	-29.79	46.91	639.1	1/12
Cell2	22.52	-69.37	-39.70	63.14	2539.7	1/12
Cell3	34.06	-69.82	-34.64	35.71	870.4	1/12
Cell4	26.77	-68.18	-34.21	54.63	1866.5	1/12
Cell5	16.13	-70.07	-28.84	52.92	1036.4	1/12
Cell6	13.94	-70.53	-32.59	55.21	1299.4	1/12
Cell7	21.18	-71.62	-32.81	61.46	2357.8	1/12
Cell8	10.35	-72.05	-19.74	34.02	373.2	1/12
Cell9	14.80	-72.11	-38.47	13.89	-	1/12
Cell10	34.34	-71.78	-35.55	28.11	595.7	1/12
Cell11	37.49	-70.04	-35.40	35.74	1125.5	1/12
Cell12	16.11	-51.33	-28.29	33.29	1045.7	1/12

Cm: membrane capacitance;
RMP: resting membrane potential;
APthreshold: action potential threshold;
APamp: action potential amplitude, AP amplitude was measured from spike threshold to positive peak;
All the data are mean +/− SEM

Supplementary Table S2. Electrophysiological properties of CiNs in *Aldh1l1-cre* mice, related to Fig. 1 and Supplementary Fig. S7.

Striatum

Induction	Input resistance (MΩ)	Cm (pF)	RMP (mV)	APthreshold (mV)	APamp (mV)	Maximal inward currents (pA)	N
CiNs	530.8 ± 86.2	16.65 ± 1.63	-71.09 ± 1.48	-40.58 ± 1.72	52.91 ± 3.70	2840.7 ± 544.6	9/9
Cell1	392.5	19.63	-70.10	-39.18	37.81	4474.4	1/9
Cell2	391.2	19.48	-67.96	-33.42	47.79	3064.2	1/9
Cell3	805.1	19.01	-66.62	-36.53	40.99	1256.7	1/9
Cell4	887.8	12.46	-73.49	-36.53	44.86	1170.7	1/9
Cell5	463.6	23.83	-70.65	-47.00	61.92	2657.6	1/9
Cell6	599.1	20.66	-71.66	-38.85	58.88	2223.9	1/9
Cell7	444.6	11.52	-69.06	-47.58	69.03	2163.4	1/9
Cell8	743.7	9.72	-81.63	-39.86	50.20	2236.0	1/9
Cell9	49.7	13.55	-68.66	-46.26	64.73	6319.1	1/9

Cortex

Induction	Input resistance (MΩ)	Cm (pF)	RMP (mV)	APthreshold (mV)	APamp (mV)	Maximal inward currents (pA)	N
CiNs	215.8 ± 62.9	16.24 ± 2.21	-70.06 ± 3.10	-47.00 ± 3.40	63.79 ± 4.07	2216.0 ± 193.3	3/3
Cell1	210.0	13.80	-64.48	-43.33	58.93	1848.7	1/3
Cell2	109.9	14.27	-75.20	-53.80	71.87	2504.3	1/3
Cell3	327.5	20.64	-70.50	-43.88	60.58	2295.1	1/3

Cm: membrane capacitance
RMP: resting membrane potential;
APthreshold: action potential threshold;

APamp: action potential amplitude, AP amplitude was measured from spike threshold to positive peak;
All the data are mean +/− SEM.

Supplementary Table S3. Small-molecule libraries for reprogramming.

FULL NAME	ABBREVIATION	DOSE(cell)	DOSE(mouse)	SOURCE	MOLECULAR WEIGHT
DBcAMP	D	100 (μM)	300 (μM)	Santa Cruz, cat.no. sc-201567	491.37
Forskolin	F	10	300	Enzo, cat. no.BML-CN100-0100	410.50
ISX9 (Isoxazole 9)	I	40	120	Tocris, cat. no.4439	234.28
CHIR99021	C	3/20	60	WUXI APPTEC	465.34
I-BET151	B	2	6	WUXI APPTEC	415.44
Y-27632	Y	10	30	Tocris, cat. no.1254	247.34

Supplementary Materials and Methods

Cell Culture
Primary mouse astrocytes were isolated from postnatal day 0 to day 3 mice. After disinfection, the mice were decapitated. Their cerebra were transferred into pre-cooled HBSS (Gibco). Remove the meningeal and cut up the cerebral cortex into small pieces. The debris of the tissue were digested with 5 mL 0.25 % trypsin (Gibco) and 1 % DNaseI (Roche) for 15 min at 37 °C, then the digestion was stopped with 5 mL astrocyte culture medium (Dulbecco's Modified Eagle Medium/F12 medium (Gibco) containing 10 % fetal bovine serum (Gibco) and 1 % penicillin-streptomycin (Invitrogen)). The cells were centrifuged for 5 min at 1500 rpm and were suspended in culture medium, a total of 10×10^6 cells were plated onto a 10 cm culture dish for differential adhesion. Thirty minutes later, the supernatant was transferred into a 75 cm^2 culture flask. The flask was incubated at 37 °C in a 5 % CO_2 / 95 % air. Refresh the medium every 3 days. Upon reaching 100 % confluency, the cultures were shaken at 260 rpm for 18 h to remove contaminating cells.

Detailed Protocol for Chemically Induced Neurons from astrocytes
Culture Medium Preparation
Neuronal induction medium: Neurobasal (Invitrogen) with 0.5 % N2 (Invitrogen), 1 % B-27 (Invitrogen), 1 % GlutaMAXTM-I (Invitrogen), 1 % penicillin-streptomycin (Invitrogen), bFGF (100 ng/mL; Origene). The small molecules were as follows: DBcAMP, 100 μM; Forskolin, 10 μM; ISX9, 40 μM; CHIR99021, 20 μM; I-BET151, 2μM and Y-27632, 10 μM.
Neuronal maturation medium: Neurobasal (Invitrogen) with 0.5 % N2 (Invitrogen), 1 % B-27 (Invitrogen), 1 % GlutaMAXTM-I (Invitrogen), 1 % penicillin-streptomycin (Invitrogen), 10 ng/mL bFGF, 20 ng/mL BDNF, 20 ng/mL GDNF. The small molecules were as follows: 10 μM Forskolin, 3 μM CHIR99021.
Small molecules used in this process are listed in Table S3.

Chemical Induction of neurons
1. Thaw matrigel (growth factors reduced; BD Biosciences) on ice according to the manufacturer's instruction and dilute it in pre-cold PBS with a ratio of 1: 30.
2. Add diluted matrigel to 6-well plates to cover the entire growth surface of the plates and keep the plates in 37 °C for 2 h to be ready to use.
3. Dissociate primary TauEGFP astrocytes or Gfap-Cre/Rosa26-tdTomato astrocytes with 0.25 % trypsin and neutralized with astrocyte culture medium.
4. Resuspend the cells with astrocyte culture medium and seed them into the matrigel-coated plates at a density of 100,000 cells per well of a 6-well plate. Cells were grown in DMEM/F-12 (Invitrogen) medium supplied with 10 % fetal bovine serum for 5-7 days until reaching 100 % confluency and the medium was refreshed every 4 days.
5. Change the medium into neuronal induction medium (day 0) and the medium was refreshed every 4 days during the chemical induction period.

Maturation of CiNs
After chemical treatment for about 16 days (the induced cells become TauEGFP-positive), the induced cells were progressed for further maturation: Replate and co-culture directly with primary wildtype mouse astrocytes.
1. Wildtype astrocytes, isolated from the postnatal C57BL/6 mice were plated at a density of 100,000 cells per well of a 6-well plate and cultured to 100 % confluency before co-cultured with induced cells.
2. Dissociate the induced cells gently by using accutase for 15 min at 37 °C and neutralize with astrocyte culture medium.
3. Centrifuge for 3 min at 1000 rpm at room temperature.
4. Carefully discard the supernatant, gently resuspend the cells with maturation medium and replate them at a density of 200,000 cells per well of a 6-well plate to co-culture with pre-existing

primary wild-type astrocytes.

5. Replated cells are co-cultured for about 14 days to become functional mature. Maturation medium was changed every 4 days.

Primary Neuron induction

Primary neurons were isolated from E18.5 wild-type ICR embryos. The hippocampus was dissected in pre-cooled HBSS (9.5 g/L Hanks' balanced salts, 4.2 mM $NaHCO_3$, 10 mM HEPES, 12 mM $MgSO_4$, 7 g/L glucose, 0.3 g/L bull serum albumin, and 0.5 % penicillin/streptomycin, with the pH adjusted to 7.4 with NaOH). Tissues were digested with 4 ml 0.25% trypsin (Invitrogen) for 5 min, and the digestion reaction was stopped with 1 mg/ml trypsin inhibitor (Sigma, dissolved in HBSS). The cells were washed three times and centrifuged for 10 min at 1,000 rpm. The neurons were resuspended in Neurobasal (Invitrogen) medium containing 2% B27 supplement and 0.5% penicillin/streptomycin. A total of $7{\sim}10{*}10^5$ cells were plated onto a well of 6-well plates that had been pre-treated with 12.5 μg/ml poly-D-lysine (Sigma). The plates were incubated at 37 °C in a 5 % CO_2 / 95 % air incubator. After 4 hours, change the fresh Neurobasal (Invitrogen) medium supplied with 2% B27 supplement, 2 mM glutamine and 0.5 % penicillin/streptomycin. Neurons were grown for 4 ~ 7 days and half of the medium was changed every three days. For induction, change the medium into neuronal induction medium (day 0) and the medium was refreshed every 3 days during the chemical induction period.

Immunostaining

Mice were anesthetized and perfused with 0.9 % saline solution followed by 4 % ice-cold PFA. Brains were removed and post-fixed in 4 % PFA overnight, then sequentially into 20 % and 30 % sucrose to cryoprotect the tissues. After embedding with OCT solution (Tissue-Tek), coronal sections were cut on a freezing microtome at a thickness of 15 μm and stored at -20 °C. TUNEL staining was performed as described in the manual of TUNEL apoptosis detection kit (Yeassen).

Immunofluorescence on cells or brain slices were all carried out as previously described (*10*). Primary antibodies included those specific to TUJ1 (Covance, rabbit anti, 1:500), MAP2 (Sigma-Aldrich, mouse anti, 1:500), SYN1 (Synaptic System, guinea pig anti, 1:100), GFP (Abcam, chicken anti, 1:200), NEUN (Millipore, guinea pig anti, 1:1000), NEUN (Abcam, rabbit anti, 1:500), GAD67 (Invitrogen, mouse anti, 1:400), vGLUT2 (Millipore, guinea pig anti, 1:400), GFAP (Agilent, rabbit anti, 1:500), S100B (Sigma-Aldrich, mouse anti, 1:1000), IBA1 (Abcam, goat anti, 1:500), MBP (Abcam, rabbit anti, 1:200), NESTIN (R&D System, mouse anti, 1:500), VIMENTIN (phosphor S55) (Abcam, mouse anti, 1:500), DARPP32 (Abcam, rabbit anti, 1:500), NPY (Abcam, rabbit anti, 1:500), PVALB (Abcam, rabbit anti, 1:500), TH (Abcam, rabbit anti, 1:500), HB9 (Developmental Studies Hybridoma Bank, mouse anti, 1:50), CTIP2 (Abcam, rat anti, 1:100) and TBR1 (Abcam, rabbit anti, 1:100). Secondary antibodies used in this study including: Alexa 405-conjugated secondary antibodies, Alexa 488-conjugated secondary antibodies, Alexa 555-conjugated secondary antibodies and Alexa 647-conjugated secondary antibodies (Invitrogen).

Electrophysiology

Cultured CiNs electrophysiology

Cells for recording were preselected based on morphological criteria typical for neurons such as the presence of projections and a non-flattened cell body. Inward currents and action potentials were recorded with the patch-clamp technique using the whole-cell configuration. For whole-cell patch-clamp recordings, the chamber was perfused with the aCSF, containing (all in mM) 141 NaCl, 2.5 KCl, 1.3 $MgCl_2$, 2.4 $CaCl_2$, 1.25 NaH_2PO_4, 10 glucose and 10 HEPES, and the pH was adjusted to 7.4 with NaOH. Action potentials were elicited by step-depolarized currents ranging from -100 pA to 20 pA at 20 pA increments. The currents were evoked by voltage steps ranging from -20 mV to 70 mV at 10 mV increments and 1 s in duration.

Brain slices electrophysiology

Brain slice preparation. The mouse brains were dissected and placed in ice-cold oxygenated aCSF containing the following (in mM): 125 NaCl, 2.5 KCl, 2 $CaCl_2$, 2 $MgCl_2$-6H_2O, 1.25 NaH_2PO4-2H_2O, 25 $NaHCO_3$ and 10 glucose, at pH 7.4. Coronary slices of 300 um thick were made with a vibratome (VT-1200S; Leica) and were maintained in an incubation chamber with oxygenated (95 % O2 / 5 % CO2) aCSF at 37 °C for 15 min and then transferred to room temperature for 30 min before being transferred to the recording chamber.

Electrophysiological recordings on brain slices

Whole-cell recordings were obtained from astrocytes, CiNs and endogenous neurons under a ×40 water-immersion objective of an Olympus BX51WI microscope (Olympus, Allentown, PA, USA). All the cells were detected in the area of about 200 um surrounding the injection cannula. Patch pipettes were pulled (∼6 MΩ tip resistance) with a P97 micropipette puller (Sutter Instruments) filled with internal pipette solution contained (in mM) 140 potassium gluconate, 1 $CaCl_2$, 10 EGTA, 2 $MgCl_2$, 5 Na_2ATP, and 10 HEPES, the pH was adjusted to 7.4 with KOH. Patch-clamp recordings were taken using an EPC-10 amplifier (HEKA) with PatchMaster. Series resistance was in the order of 10~30 MΩ, which was compensated by 60–80% during the experiments. Recordings were discarded if the series resistance increased by more than 20% during the course of the recordings. In whole-cell current-clamp recordings (with an Axon 700B amplifier, Molecular Devices, San Jose, CA, USA), A 100 ms, 20 pA test pulse was applied to the recording cells right after breaking into the whole-cell configuration. A fast-rising component and a slow-rising component of voltage response were clearly visible. Then zoomed into the fast-rising component of the voltage responses and turned up the pipette capacitance neutralization slowly to shorten the rise time of the fast-rising component until the oscillations of voltage responses appeared. Subsequently, the capacitance compensation was decreased just until the oscillations disappeared. For bridge balance of current-clamp recordings, the value of bridge balance was increased slowly until the fast component of the voltage response disappeared, and the slow-rising component appeared to rise directly from the baseline. Series resistance and pipette capacitance were compensated using the bridge balance and pipette capacitance neutralization options in the Multiclamp 700B command software. The bridge balance value was between 20 and 30 MΩ and the pipette capacitance neutralization value was between 3 and 5 pF. Electrophysiological recordings were made using a Multiclamp 700B amplifier (Molecular Devices, Sunnyvale, CA, USA). Recordings were filtered at 10 kHz and sampled at 50 kHz. All recordings were conducted at room temperature.

For current-clamp recordings, a hyperpolarized current was injected into the cells to membrane potentials around -70 mV. Single action potential was elicited by input currents with sweep intervals of 1 s. Continuous action potentials were elicited by step-depolarized currents ranging from -200 pA to 400 pA at 50 pA increments with sweep intervals of 5 s. For detecting inward currents, the membrane potential was held at -70 mV under voltage-clamp mode. The currents were evoked by voltage steps ranging from -20 mV to 100 mV at 10 mV increments and 400 ms in duration. For recording spontaneous EPSCs (Excitatory Post Synaptic Currents), cells were voltage clamped to -70 mV over 10 min. Recordings were filtered at 10 kHz and sampled at 50 kHz.

Data Analysis

Data were acquired and analyzed using pCLAMP 10.0 software and Origin 8.5 software (OriginLab Corp., Northampton, MA, USA). The cells were held at 0 pA to record the resting membrane potentials (RMP). Action potential amplitude was measured relative to the threshold. The maximal inward currents were measured at maximal inward current amplitude in all test voltage step. The input resistance was calculated according to Ohm's law ($V = IR$) by measuring the steady-state (post-sag) Vm deflection generated by −100 pA square current (400 ms). After hyperpolarization (AHP) was measured from the most negative membrane potential during the repolarization process relative to the RMP or −70 mV. Sampling frequencies: Injection current duration time was divided by AP numbers.

Quantification and Statistical Analysis

All quantifications of images were conducted using Las X or ImageJ software.

For *in vitro* conversion efficiency analysis and lineage tracing analysis (Supplementary Figs. S1-2), images were randomly selected from independent batches of cell cultures for quantification.

To confirm cell identity of labeled cells in vivo (Supplementary Fig. S6), at least 3 sections per mice (at least 3 mice) were analyzed to quantify percentages of cell type markers co-expression.

To systemically quantify CiNs in Aldh1l1-cre mice (Fig.1c), 5 brain sections at 45-μm intervals around the infusion core per mouse were analyzed. Conversion efficiencies of CiNs were quantified as percentage of $NEUN^+/EGFP^+$ cells out of $EGFP^+$ cells within the ROI (r = 300 μm). For each mouse, section with most CiNs generated were used for efficiency calculation. When studying subtype specification, all $NEUN^+/EGFP^+$ cells in 5 brain sections per mouse were analyzed (Fig. 2b).

For dosage optimization, single small molecule omission analysis (Supplementary Fig. S4), cell counting area was selected surrounding the injection site: upper edge was extended from the needle hole to the corpus callosum (Cc); the bottom, left and right edges were the outward extensive zone of 100 μm from the astrocytes reactivated area. The number of CiNs were obtained by counting the number of $NeuN^+/tdTomato^+$ cells on one section at the infusion core. Three mice were used for each analysis.

For the RABV tracing analysis (Fig. 4c), the numbers of $EGFP^+/DsRed^+$ cells in every section along the injection area was counted. Only cell bodies in which the nucleus was clearly visible were counted.

To quantify microglial response (Supplementary Fig. S5c), one brain section at the infusion core per mouse was used and the percentage of IBA1 fluorescent area was analyzed automatically by ImageJ software[1]. Firstly, convert a picture to 8 bits. Then set a ROI (a circle r = 600 μm around the center of the cannula, exclude SVZ, CC, and cortex) above the threshold (10%) per section. Finally, measure the area fraction within the selected ROI.

All statistical tests were performed with GraphPad Prism 7.0 Software. Statistical significance was defined at $*p < 0.05$, $**p < 0.01$, $***p < 0.001$, and $****p < 0.0001$. Unpaired two-tailed t tests were used for comparisons between two groups. One-way ANOVA was used followed by Tukey's Multiple Comparison test for comparisons between three or more groups. Values are presented as mean ± SEM. All biological replicates (n) are derived from at least three independent experiments.

RNA sequencing

The sequencing data quality were checked by FastQC (version 0.11.8). RNA-seq reads from each sample were aligned to mouse reference genome mm10 by using TopHat2[2] (version 2.1.1) with default parameters. Gene expression levels (FPKM values) were quantified by Cuffdiff[3] (version 2.2.1). For differential gene expression analysis, reads count of each gene were obtained by HTSeq[4] (version 0.11.1).

Genes had average expression values greater than zero were considered as valid. The pair-wise differential gene expression analysis at different time points were performed by R package edgeR[5] (version 0.38.0). Genes with absolute value of leading log-fold-change greater than 2 and FDR < 0.01 defined as differential expressed genes. PCA and clustering the PCA results were performed by R package FactoMineR[6] (version 2.0) function "PCA" and "HCPC" separately. Hierarchical clustering was performed by R function "hclust". Heatmap were plot by R package pheatmap (version 1.0.12). GO analysis were performed by clusterProfiler[7] (version 3.14.0). The mouse transcription factor list were downloaded in AnimalTFDB[8] (version 3.0). The gene regulation network was built by R package GENIE3[9] (version 1.8.0). Network visualization were performed by Cytoscape[10] (version 3.7.2). R(version 3.6.0) were used for gene expression analysis.

Transcriptome analysis of CiNs and primary neurons
To compare gene expression profile among reprogramming neurons with primary neurons, differential genes among D0 and primary neurons (PN) with which absolute value of leading log-fold-change greater than 4 and FDR < 0.01 were used to build hierarchical clustering trees.

Single cell RNA-sequencing analysis
Library construction and sequencing
We converted the single-cell RNA collected from the *in vivo* patch-clamped eGFP^{+} cells to cDNA for generating the sequencing libraries following the protocol of Smart-seq2[11,12]. After isolation of single chemical induced neuron from striatum, the single cells were picked into 0.3ul electrode fluid by mouth pipette then blow into 4 ul lysis buffer. Lysis buffer solution containing: 1 U/ml RNase inhibitor, 0.1% Triton X-100, 2.5 mM dNTP, 25 mM Oligo dT30VN ,5mM dNTP and about 30,000 molecular number of ERCC. After incubation at 72℃ for 3 min, the cDNAs were synthesized by adding 5.7 ul solution containing: 17.54 U/ul SuperScript II reverse transcriptase, 1.75 U/ul RNase inhibitor, 1.75×Superscript II first-stranded buffer, 8.78mM DTT, 1.75 M Betaine, 10.52 mM MgCl2 and 1.75 mM TSO primer, and incubated at 25℃ for 5 min, 42℃ for 1.5 hr, 11 cycle of 50℃ for 2 min and 42℃ for 2 min, then 72℃ for 15 min and held at 4℃. The double stranded cDNAs were amplified with solution containing: 1.67× KAPA HiFi HotStart Ready Mix, 0.17 mM IS PCR following the PCR procedure of 95℃ for 3 min, 4 cycles of 98℃ for 20 s,65℃ for 30 s and 72℃ for 5 min, then 16 cycles of 98℃ for 20 s, 67℃for 15 s and 72℃ for 5 min, 72℃ for 5 min, and held at 4℃. Pull 25ul the amplified cDNAs together, purify with 0.8× XP beads (Beckman) twice. Finally, we used 25 ng cDNA for the library constructing by KAPA Hyper Prep Kit and sequenced on Illumina HiSeq 4000 platform.

Read processing and quantification of gene expression
Adapters and low-quality reads were trimmed using Python script AfterQC[13]. Paired-end reads were aligned to the reference genome GRCm38 primary assembly[14] downloaded from ensemble using STAR (STAR 2.5.3a)[15] with default settings. Then Reads were counted using featureCounts (featureCounts 1.5.3)[16]. The R package, scater (Single-cell analysis toolkit for gene expression data in R, version 1.6.3)[17], was employed for quality control, normalization and data visualization. Gene expression was normalized to the number of fragments per kilobase million (FPKM value)[18]. The raw dataset contained 45900 genes in total 59 samples.

Quality control
We removed genes that did not have an expression value of at least 1 FPKM across all samples. The filtered dataset contained 20740 genes in total 59 samples.

We use the MAD-based definition of outliers to remove putative low-quality cells from the dataset. The filter was 3 times lower than the median MAD. One cell was removed by the library size filter and five cells with 3 times lower than the ERCC proportion median MAD. The dimensionality of the dataset was then reduced from 59 cells to 53 cells.

Filter out low-abundance genes which defined as those with an average count below a filter threshold of 1. Removal of these genes, there remains 14643 genes. We distinguished biological components with technical components by fitting a mean-dependent trend to the variances of the log-expression for the ERCC transcripts. HVGs were defined as genes with biological component greater than or equal to 0.5 and a false discovery rate (FDR) of 5%. Identify correlated gene pairs with Spearman’s rho using the correlatePairs function. We visualize the expression profiles of the correlated HVGs with a heatmap and saved to file for future reference.

Dimensionality reduction techniques
We performed denoisePCA unction to remove technical noise from expression, using 5 principle component for dimensionality reduction. Using this function, the remaining PCs can show more differences in biological factors, which can improve the accuracy of subsequent clustering. The t-

sochastic neighbour embedding (t-SNE) method[19] was used to separate cells into clusters in the t-SNE plot.Clusters are defined by cutting the dendrogram with cutree function from R package dynamic tree cuts[20].

Differential gene expression analysis
Differential gene expression analysis was performed using findMarkers function. A heatmap of gene expression from 1056 DEGs was generated using plotHeatmap from R package scater. Enriched Gene Ontology (GO) analysis of DEGs from every cluster was performed using the online tool, Metascape [21].

Neuron gene score analysis
We selected 211 well-known neuronal marker genes from the online database[22]. Preserve genes that have non-zero counts in at least 25 cells. Scale the Average of the normalized gene expression of neuron bio-markers, respectively using the scale function of base R packages.

Pan neuron function gene heatmap
Based on the differentially expressed genes GO trems of cluster3, combining with previous studies, we screened out pan neuron-related genes and neuron subtype genes to form heatmap plot.

Data Resources
The accession number for the RNA sequencing data reported in this study is NCBI GEO: GSM144993.

References:

1 Gao, J. *et al.* Human Neural Stem Cell Transplantation-Mediated Alteration of Microglial/Macrophage Phenotypes after Traumatic Brain Injury. *Cell Transplant* **25**, 1863-1877, doi:10.3727/096368916x691150 (2016).
2 Kim, D. *et al.* TopHat2: accurate alignment of transcriptomes in the presence of insertions, deletions and gene fusions. *Genome Biol* **14**, R36, doi:10.1186/gb-2013-14-4-r36 (2013).
3 Trapnell, C. *et al.* Differential analysis of gene regulation at transcript resolution with RNA-seq. *Nat Biotechnol* **31**, 46-53, doi:10.1038/nbt.2450 (2013).
4 Anders, S., Pyl, P. T. & Huber, W. HTSeq--a Python framework to work with high-throughput sequencing data. *Bioinformatics* **31**, 166-169, doi:10.1093/bioinformatics/btu638 (2015).
5 Robinson, M. D., McCarthy, D. J. & Smyth, G. K. edgeR: a Bioconductor package for differential expression analysis of digital gene expression data. *Bioinformatics* **26**, 139-140, doi:10.1093/bioinformatics/btp616 (2010).
6 Lê, S., Josse, J. & Husson, F. FactoMineR: An R Package for Multivariate Analysis. *Journal of Statistical Software* **25**, 18, doi:10.18637/jss.v025.i01 (2008).
7 Yu, G., Wang, L. G., Han, Y. & He, Q. Y. clusterProfiler: an R package for comparing biological themes among gene clusters. *OMICS* **16**, 284-287, doi:10.1089/omi.2011.0118 (2012).
8 Hu, H. *et al.* AnimalTFDB 3.0: a comprehensive resource for annotation and prediction of animal transcription factors. *Nucleic Acids Res* **47**, D33-D38, doi:10.1093/nar/gky822 (2019).
9 Huynh-Thu, V. A., Irrthum, A., Wehenkel, L. & Geurts, P. Inferring regulatory networks from expression data using tree-based methods. *PloS one* **5**, doi:10.1371/journal.pone.0012776 (2010).
10 Shannon, P. *et al.* Cytoscape: a software environment for integrated models of biomolecular interaction networks. *Genome research* **13**, 2498-2504, doi:10.1101/gr.1239303 (2003).
11 Liu, J. *et al.* Functional In vivo Single-cell Transcriptome (FIST) Analysis Reveals Molecular Properties of Light-Sensitive Neurons in Mouse V1. *bioRxiv*, 382002 (2018).
12 Picelli, S. *et al.* Full-length RNA-seq from single cells using Smart-seq2. *Nature protocols* **9**, 171 (2014).

13 Chen, S. *et al.* AfterQC: automatic filtering, trimming, error removing and quality control for fastq data. *BMC bioinformatics* **18**, 80 (2017).
14 Kersey, P. J. *et al.* Ensembl Genomes 2018: an integrated omics infrastructure for non-vertebrate species. *Nucleic acids research* **46**, D802-D808 (2018).
15 Dobin, A. *et al.* STAR: ultrafast universal RNA-seq aligner. *Bioinformatics* **29**, 15-21 (2013).
16 Liao, Y., Smyth, G. K. & Shi, W. featureCounts: an efficient general purpose program for assigning sequence reads to genomic features. *Bioinformatics* **30**, 923-930 (2014).
17 McCarthy, D. J., Campbell, K. R., Lun, A. T. & Wills, Q. F. Scater: pre-processing, quality control, normalization and visualization of single-cell RNA-seq data in R. *Bioinformatics* **33**, 1179-1186 (2017).
18 Liu, J. *et al.* The primate-specific gene TMEM14B marks outer radial glia cells and promotes cortical expansion and folding. *Cell Stem Cell* **21**, 635-649. e638 (2017).
19 Hinton, L. v. d. M. a. G. Visualizing Data using t-SNE. *Journal of Machine Learning Research* **9**, 2579-2605 (2008).
20 Langfelder, P., Zhang, B. & Horvath, S. dynamicTreeCut: Methods for detection of clusters in hierarchical clustering dendrograms. *R package version*, 1.63-61 (2014).
21 Zhou, Y. *et al.* Metascape provides a biologist-oriented resource for the analysis of systems-level datasets. *Nature communications* **10**, 1-10 (2019).
22 Franzén, O., Gan, L.-M. & Björkegren, J. L. PanglaoDB: a web server for exploration of mouse and human single-cell RNA sequencing data. *Database* **2019** (2019).
